# High Serum Asprosin Levels Are Associated with Presence of Metabolic Syndrome

**DOI:** 10.1155/2021/6622129

**Published:** 2021-03-02

**Authors:** Tao Hong, Jiao-Yang Li, Ya-Di Wang, Xiao-Yan Qi, Zhe-Zhen Liao, Poonam Bhadel, Li Ran, Jing Yang, Bin Yan, Jiang-Hua Liu, Xin-Hua Xiao

**Affiliations:** Department of Metabolism and Endocrinology, The First Affiliated Hospital of University of South China, Hengyang 421001, Hunan, China

## Abstract

**Objective:**

Asprosin, a new adipocytokine, has reportedly been associated with glucose release, dyslipidemia, and insulin resistance (IR). However, the relationship of asprosin with metabolic syndrome (MetS) remains unknown. This study aimed to investigate serum asprosin levels in MetS as well as their association with various metabolic parameters in humans.

**Methods:**

A total of 131 consecutive patients with MetS, and 162 age-matched, healthy subjects were recruited for this study. Serum asprosin concentrations were determined using the enzyme-linked immunosorbent assay. Lipid profile, glucose, insulin, and inflammatory markers were also measured.

**Results:**

Serum asprosin levels were higher in subjects with MetS (23.52 [16.70, 32.05] ng/mL) than in controls (16.70 [12.87, 22.38] ng/mL; *P* < 0.01), and they showed an increasing trend with increasing numbers of metabolic components (*P* for trend < 0.01). In all studied subjects, serum asprosin levels were positively correlated with body mass index, waist circumference, triglycerides, fasting plasma glucose, 2-hour plasma glucose, fasting insulin, homeostatic model assessment of insulin resistance (HOMA-IR) index, interleukin-6, and monocyte chemoattractant protein-1 and negatively correlated with high-density lipoprotein cholesterol (*P* < 0.05). In multiple linear regression, asprosin was independently and positively correlated with triglyceride and HOMA-IR (*P* < 0.05). Binary logistic regression revealed that asprosin was independently and positively correlated with the occurrence of MetS and IR, even after controlling for anthropometric variables, lipid profiles, and inflammatory markers.

**Conclusion:**

Asprosin is a potential metabolic-related adipokine and may be related to IR and MetS. This trial was registered with ChiCTR, ChiCTR1800018347.

## 1. Introduction

Metabolic syndrome (MetS) is a constellation of clinical features that include insulin resistance (IR), obesity, dyslipidemia, hyperglycemia, and elevated blood pressure [1]. A meta-analysis has revealed that MetS is associated with an approximate doubling of cardiovascular disease risk and a 1.5-fold increased risk of all-cause mortality [2]. Given the rapidly increasing prevalence of MetS (20–30% of the general world population) and its possible harmful outcome [3, 4], further research is imperative to reveal its predictive factors and mechanisms. In recent years, some adipocyte-related cytokines have proven to be MetS-related biomarkers, such as adiponectin, zinc-*α*2-glycoprotein, and betatrophin [5–7], and increasing evidence demonstrates that cytokines play an important role in the pathogenesis of MetS.

Asprosin, a 140-amino-acid C-terminal profibrillin, is a recently discovered fasting-induced glucogenic adipokine, which was initially detected in neonatal progeroid syndrome [8]. Asprosin is mainly secreted into the blood by white adipose tissues, targeting the liver and exerting a glucogenic effect through OR4M1, an olfactory G-protein-coupled receptor [8]. It also leads to impairment of insulin sensitivity and secretion through PKC*δ*-activated endoplasmic reticulum stress and TLR4/JNK-mediated inflammation pathways [9, 10].

It has been highlighted that elevated asprosin levels were observed in humans and mice with IR, type 2 diabetes mellitus (T2DM), or obesity [8, 11–16]. A single injection of asprosin caused an immediate spike in blood glucose and insulin in mice models and humans [8]. Conversely, genetic deficiency and specific antibody-targeting asprosin result in improved insulin sensitivity as well as reduced appetite and body weight [8, 17]. These data indicated that asprosin may serve as a novel therapeutic target for IR-related diseases. However, findings regarding the relation between serum asprosin, adults with polycystic ovary syndrome (PCOS), and children with obesity are inconsistent. Some studies have suggested reduced levels [18], and others have indicated elevated levels [19–22], whereas a number did not find significant changes [23]. As MetS is strongly associated with glucose metabolism, IR, and inflammation, we hypothesized that abnormal regulation of asprosin could be associated with the manifestation of MetS. Therefore, we conducted a cross-sectional study to measure serum asprosin levels in MetS as well as analyze their association with various metabolic parameters.

## 2. Materials and Methods

### 2.1. Research Objects

A total of 293 subjects (including 131 MetS patients and 162 age-matched, healthy controls) who had undergone routine physical examinations at the First Affiliated Hospital of University of South China from February 2018 to December 2018 were recruited for this study. MetS was defined according to the criteria set by a joint statement of the International Diabetes Federation Task Force on Epidemiology and Prevention [1]. Subjects who fulfilled at least three of the following five criteria were defined as having MetS: (1) waist circumference ≥80 cm in women or 85 cm in men; (2) high-density lipoprotein cholesterol (HDL-C) ≤1.3 mmol/L in women or ≤1.0 mmol/L in men; (3) triglycerides (TGs) ≥1.7 mmol/L; (4) blood pressure ≥130/85mmHg or current use of antihypertensive drugs; and (5) fasting plasma glucose (FPG) ≥5.6 mmol/L (100 mg/dL), previous diagnosis of type 2 diabetes, or use of antidiabetic medication (insulin or oral agents). Age-matched healthy subjects without clinical evidence of major diseases were recruited as the controls. All participants completed a uniform questionnaire containing demographics, medical history, recent medication history, and lifestyle factors (smoking and alcohol). MetS and healthy individuals had not been treated with any medicine, including hypoglycemic and lipid-lowering agents, as well as diet modification or exercise. Exclusion criteria included the following: (1) subjects aged ≤18 and ≥70; (2) subjects suffering from any kind of infection; (3) a history of cardiovascular disease; (4) acute or chronic complications; (5) heart, liver, or kidney failure; (5) pregnancy; or (6) other known major disease. This study was approved by the Ethics Committee of the First Affiliated Hospital of University of South China, following the principles of the Helsinki Declaration. Furthermore, all subjects provided written informed consent.

### 2.2. Anthropometric and Biochemical Evaluation

Measurement parameters, including height, weight, waist circumference, and blood pressure (systolic blood pressure (SBP) and diastolic blood pressure (DBP)), were measured using a standardized protocol. An analyzer of bioelectrical impedance was used to measure the percentage of body fat (Fat%). Body mass index (BMI) was defined as the individual's body weight (kg) divided by the square of their height (m).

Blood samples were obtained after fasting overnight for at least 10 h. Total cholesterol, TG, low-density lipoprotein cholesterol (LDL-C), HDL-C, fasting blood glucose (FBG), 2-hour plasma glucose (2h-PG), glycosylated hemoglobin, and fasting insulin (FIns) were determined as previously published [24]. The homeostasis model assessment of the insulin resistance (HOMA-IR) index was calculated as follows: FPG (mmol/L) × FIns (*μ*U/mL)/22.5.

### 2.3. Measurement of Adipokines

Routine laboratory tests were performed in the accredited central laboratory of the hospital according to standard protocols. Serum was obtained after centrifugation, aliquoted, and then stored at –80°C in preparation for the enzyme-linked immunosorbent assay (ELISA). Serum asprosin levels were measured using the commercial Sandwich ELISA kits from Abbexa Ltd. (Cambridge, UK, Catalog no. abx257694). Serum interleukin (IL)-6 and monocyte chemoattractant protein (MCP)-1 levels were measured using ELISA kits from R&D Systems, Inc., (Minneapolis MN, USA, Catalog nos. DCP00 and D6050). The average intra- and interassay coefficients of variation were 10% and 6% for asprosin, 7.8% and 6.7% for MCP-1, and 4.2% and 6.4% for IL-6, respectively. The samples derived from both the control and MetS subjects underwent the same processing and the same number of freeze-and-thaw cycles. Measurements were performed following manufacturer instructions. All samples were assayed in duplicate and random order.

### 2.4. Statistical Analysis

The data were presented as mean ± SD or median with interquartile range. Normal distribution of the data was determined using the *Kolmogorov–Smirnov* test. Variables not normally distributed were logarithmically transformed to near normality before analysis. Comparisons of categorical and continuous variables were performed using the chi-squared and one-way ANOVA tests, respectively. Correlations between variables were assessed using the *Pearson* correlation analysis by controlling for the covariates. The independent associations between asprosin and variables were determined using multiple linear regression. The adjusted odds ratios and 95% confidence intervals for asprosin levels and MetS or IR were presented using binary logistic regression. The receiver operator characteristic (ROC) curve was calculated to identify the abilities of asprosin to predict MetS and IR. The trends of the association between asprosin levels and MetS were analyzed using the *row mean scores* test and *Cochran–Armitage* trend tests. The multiple imputation procedure was performed (five imputations) to impute missing data for covariates. *P* < 0.05 was considered statistically significant.

The *post hoc* power analysis of sample size was evaluated, taking the serum asprosin levels in subjects with MetS and in controls as the evaluation indicator. The data were logarithmically transformed to near normality before analysis. The control group (l g (asprosin)) = 1.226 ± 0.187 ng/mL, MetS group (l g (asprosin)) = 1.378 ± 0.212 ng/mL, population standard deviation (*σ*) = 0.212, and power (1−*β*) was set at 0.90 and *α* = 0.05 (two side). Sample size was calculated using PASS 15.0.5 software. In total, 293 subjects were enrolled in our study. The sample size was considered adequate.

## 3. Results

### 3.1. Characteristics and Serum Asprosin Levels of the Subjects

Anthropometric, biochemical, and metabolic parameters of the 293 subjects are shown in Table 1. Age and sex were comparable between controls and MetS subjects. As expected, MetS patients had significantly higher levels of BMI, waist circumference, fat%, SBP, DBP, TG, FIns, FPG, 2h-PG, HOMA-IR, IL-6, and MCP-1 and lower HDL-C than those of the controls (*P* < 0.01 or *P* < 0.05). However, there were no significant differences in recent smoking (%), TC, and LDL-C between control and MetS groups (*P* < 0.05).

**Figure 1:**
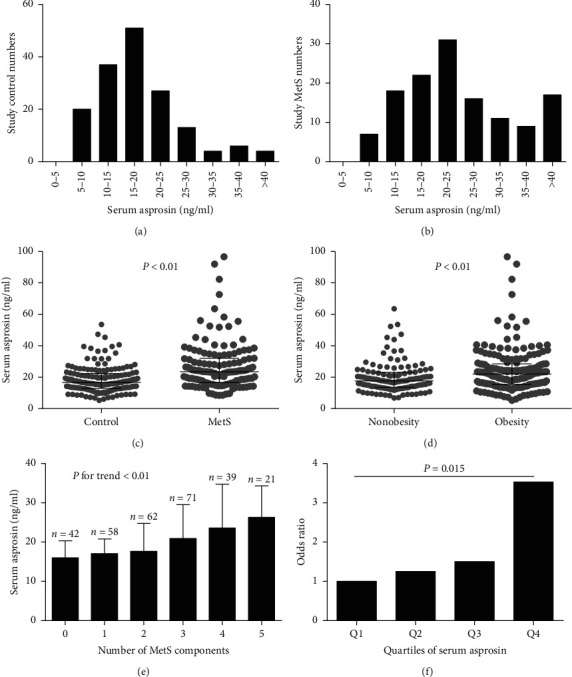
Serum asprosin levels in the study population. (a) Distribution of serum asprosin levels in 162 control subjects; (b) distribution of serum asprosin levels in 131 MetS subjects; (c) serum asprosin levels in control and MetS subjects; values were provided as median with interquartile range and log-transformed before analysis; (d) serum asprosin levels in the whole study population according to waist circumference (nonobesity: waist circumference < 85 cm in men or 80 cm in women and obesity: waist circumference ≥ 85 cm in men or 80 cm in women); values were provided as median with interquartile range and log-transformed before analysis; (e) serum asprosin levels increased progressively with increasing numbers of MetS components; values were provided as median with interquartile range and log-transformed before analysis; (f) odds ratios for having MetS according to the quartiles of serum asprosin levels (reference, the lowest quartile); Q1: <14.21 ng/mL; Q2: 14.21–19.26 ng/mL; Q3: 19.26–25.79 ng/mL, and Q4: >25.79 ng/mL (Q: quartile).

The distribution of serum asprosin is displayed in Figure 1(a) for controls and Figure 1(b) for MetS subjects. Serum asprosin concentrations ranged from 7.04 to 40.52 ng/mL in 95% of the controls and 8.84 to 79.41 ng/mL in 95% of MetS subjects. Importantly, serum asprosin levels were significantly higher in MetS subjects than in controls (23.52 [16.70, 32.05] *vs.* 16.70 [12.87, 22.38] ng/mL, *P* < 0.01; Figure 1(c)), and increased in a stepwise fashion as the number of MetS components increased (*P* for trend < 0.01, Figure 1(e)). Furthermore, subjects with abdominal obesity had significantly higher serum asprosin levels than those in subjects without (*P* < 0.01, Figure 1(d)). However, there was no sex difference in serum asprosin between the control and MetS participants (*P* > 0.05), indicating that asprosin has no distinct sexual dimorphism.

### 3.2. Correlation of Asprosin with Clinical Parameters in Study Subjects

Serum asprosin concentrations were positively correlated with adiposity-related parameters (BMI and waist circumference, *P* < 0.01), an adverse lipid profile (increased TG and decreased HDL-C, *P* < 0.01), parameters of blood glucose (FPG and 2h-PG, *P* < 0.01), insulin resistance indices (FIns and HOMA-IR, *P* < 0.01), and inflammatory markers (MCP-1 and IL-6, *P* < 0.01) (Table 2). After further adjustment for age and BMI, all these correlations remained similar, except for waist circumference and MCP-1. Multiple regression analyses of stepwise models were performed to determine which variables were independently associated with serum asprosin concentrations. Only TG and HOMA-IR were independently related to serum asprosin (*P* < 0.05), with a multiple regression equation of *Y*_(lg asprosin)_ = 1.163 + 0.033 × TG + 0.028 × HOMA-IR (*R*^2^ = 0.131) (Table 2).

### 3.3. The Effect of Serum Asprosin on the Incidence of MetS and IR

**Figure 2:**
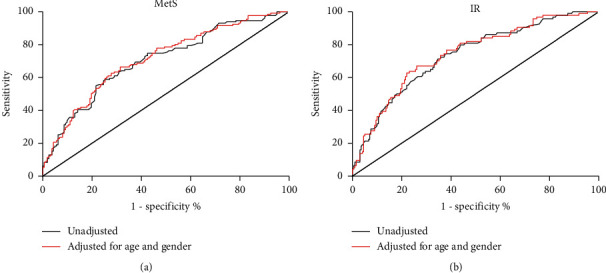
ROC curve analyses ROC curve analyses were performed for the prediction of serum asprosin for 16 MetS (a) and IR (b). MetS, metabolic syndrome; IR, insulin resistance.

Serum asprosin concentrations were markedly related to MetS and IR, even after adjustment for age, sex, BMI, lipid profile, and inflammatory markers in an additive multiple logistic regression model (Table 3). To further investigate the association of asprosin with MetS, asprosin levels were categorized using their quartile values (quartile 1: <14.21 ng/mL; quartile 2: 14.21–19.26 ng/mL; quartile 3: 19.26–25.79 ng/mL, and quartile 4: >25.79 ng/mL). Logistic regression analysis was subsequently performed to calculate the odds of having MetS after controlling for the covariates. When asprosin levels were in quartile 4, the odds ratio of having MetS was 3.533 (*vs.* quartile 1, *P*=0.015; Figure 1(f)). Furthermore, a significant linear trend over increasing asprosin categories was observed for the presence of MetS by the *row mean scores* test and the *Cochran–Armitage* trend test (Table 4). Finally, ROC curve analyses revealed that the areas under the curve (AUCs) for serum asprosin (age and sex adjusted) were 0.712 and 0.742 for the prediction of MetS (Figure 2(a) and IR (Figure 2(b)), respectively.

## 4. Discussion

The main findings of this study were as follows: (i) serum asprosin levels were markedly higher in MetS patients than in healthy controls, and they showed an increasing trend with increasing numbers of metabolic components; (ii) serum asprosin levels were positively correlated with BMI, waist circumference, FPG, 2h-PG, FIns, HOMA-IR, TG, MCP-1, and IL-6 and negatively correlated with HDL-C; (iii) serum asprosin was independently and positively correlated with the occurrence of MetS and IR, even after controlling for the covariates.

Asprosin has recently been identified as a white tissue-derived novel adipokine, and its concentrations have been confirmed to be increased in adults with T2DM and those with obesity [11–16]. However, evidence for the association between asprosin and MetS status remains scarce. Here, we demonstrated that serum asprosin levels were markedly elevated in MetS, a finding that corroborated that of a recent study conducted in patients with T2DM [13–16]. Nevertheless, the reason for increased asprosin secretion is unknown. Previous studies have demonstrated that plasma asprosin was pathologically elevated in mice and humans with IR, while asprosin-specific monoclonal antibody lowered plasma asprosin and improved insulin sensitivity in these mice [8, 17]. Hence, we speculated that asprosin may serve as a risk factor associated with the pathogenesis of MetS. However, the cross-sectional nature of the current study still could not rule out the possibility that the elevation of serum asprosin in MetS might have been a compensatory upregulation for counteracting the metabolic stress produced by adiposity, hyperglycemia, or hyperlipidemia. Therefore, a follow-up study will be necessary.

IR is generally considered a root causative factor for developing MetS. Adipose tissue has the endocrine role of regulating energy balance and glucose homeostasis. Several adipose tissue-secreted cytokines can either enhance or impair insulin action [25]. Data from the current study clearly revealed that asprosin was significantly positively correlated with the well-known indices of MetS in all individuals. Among these indices, HOMA-IR was independently associated with serum asprosin levels. Previous studies have found that an intraperitoneal injection of the asprosin antibody can significantly reduce serum insulin levels and improve IR in obese mice [17]. Three recent clinical studies have also found that circulating-asprosin concentrations are positively correlated with IR in patients with T2DM or PCOS [13, 15, 19]. It is suggested that the correlation between asprosin and MetS may be partly attributed to IR. In addition, ROC curve analysis revealed that serum asprosin might have been a useful marker for the prediction of MetS and IR in our study population. On one hand, the AUC values (0.712 and 0.742) were considered moderately significant, which might have been due to the relatively small sample size and a nonnormal distribution in the study population. On the other hand, serum asprosin may not be an ideal marker for predicting MetS and IR.

Dyslipidemia and hyperglycemia are pathological states that are characteristic of MetS, and they play crucial roles in the pathogenesis of the disease [1]. Our data demonstrate that serum asprosin levels are significantly correlated with TG, HDL-C, FBG, and 2h-PG even after adjustment for age and BMI. Our multiple stepwise regression analysis identified TGs as significant, independent contributors to circulating-asprosin levels. These findings raise the hypothesis that asprosin may provide a molecular association between glucose-lipid metabolism and MetS. Although it would be premature to conclude the causal effects of asprosin on these parameters, it would be of interest to explore whether therapeutically targeting asprosin may ameliorate metabolic disorder in MetS subjects.

Chronic low-grade inflammation is closely related to obesity and IR and potentially leads to the pathogenesis of some metabolic-related diseases. Previous studies have demonstrated that asprosin promotes hepatic glucose production by activating the cAMP second-messenger system, which is also involved in the inflammatory response [8]. Another *in vitro* experiment displayed that siRNA-mediated asprosin suppression improved NF-*κ*B phosphorylation and release of TNF-*α* and MCP-1 in palmitic-treated pancreatic cells [10]. However, a recent clinical research study revealed that serum asprosin had no significant association with the high-sensitivity C-reactive protein (hs-CRP) in patients with diabetes, which functions as an acute marker of inflammation [13]. These controversial results prompted us to further explore the association between asprosin and metabolic inflammation. IL-6 and MCP-1 are important markers of chronic low-grade inflammation and are also known to play a key role in the pathogenesis of obesity and IR. Thus, we measured the levels of inflammatory markers IL-6 and MCP-1 in all subjects and discovered that, even after adjusting for age and BMI factors, asprosin was significantly correlated with IL-6. Further studies are still required to clarify the precise function of asprosin in metabolic inflammation.

This study also has certain limitations. First, it is difficult to deduce the causal relationship between serum asprosin levels and MetS due to the cross-sectional study design. Hence, a larger sample of prospective studies is required to confirm this relationship. Second, this study focuses on the Chinese population; therefore, it needs to be carefully extended to other ethnic groups. Third, related inflammation indicators, such as hs-CRP, were not assessed. Fourth, our study only detected serum levels based on ELISA, and there might have been some random measurement errors.

## 5. Conclusion

Overall, our data suggest that asprosin is a novel metabolic-regulated adipokine that is considerably associated with IR and MetS.

## Figures and Tables

**Table 1:** Clinical and biochemical characteristics of controls and MetS subjects.

Variables	Controls	MetS	*P* value
No. of subjects	162	131	—
Gender, M/F	78/84	65/66	0.802
Age (years)	49.47 ± 9.16	50.90 ± 9.45	0.236
Recent smoking (%)	18.5	26.7	0.093
BMI (kg/m^2^)	23.28 ± 2.49	25.52 ± 3.02	<0.001
Waist circumference (cm)	81.3 ± 7.5	87.2 ± 8.9	<0.001
Fat (%)	27.61 ± 5.57	29.66 ± 3.68	<0.001
SBP (mm Hg)	123.7 ± 14.2	133.4 ± 13.3	<0.001
DBP (mm Hg)	74.9 ± 8.9	82.2 ± 9.4	<0.001
TC (mmol/l)	4.73 ± 0.98	4.89 ± 1.05	0.181
TG (mmol/l)^a^	1.10 (0.82, 1.39)	1.92 (1.58, 2.49)	<0.001
LDL cholesterol (mmol/l)	2.42 ± 0.90	2.60 ± 1.04	0.115
HDL cholesterol (mmol/l)	1.57 ± 0.36	1.18 ± 0.41	<0.001
FPG (mmol/l)^a^	5.10 (4.74, 5.41)	6.26 (5.26, 8.89)	<0.001
2h-PG (mmol/l)^a^	5.82 (5.49, 6.23)	7.71 (6.14, 15.04)	<0.001
FIns (mU/l)^a^	7.20 (6.06, 8.79)	8.93 (7.78, 10.62)	<0.001
HOMA-IR^a^	1.60 (1.30, 2.06)	2.53 (1.84, 3.93)	<0.001
IL-6 (ng/l)	12.62 ± 5.20	17.02 ± 6.32	<0.001
MCP-1 (ng/l)	118.95 ± 39.49	146.94 ± 52.41	<0.001

Values were given as means ± SD or median with interquartile range. ^a^Log-transformed; MetS, metabolic syndrome; BMI, body mass index; Fat, percentage of body fat; SBP, systolic blood pressure; DBP, diastolic blood pressure; TC, total cholesterol; TG, triglyceride, LDL, low-density lipoprotein; HDL, high-density lipoprotein; FPG, fasting plasma glucose; 2h-PG, 2-hour plasma glucose; FIns, fasting insulin; HOMA-IR, homeostasis model assessment of insulin resistance; IL-6, interleukin-6; MCP-1, monocyte chemotactic protein-1.

**Table 2:** Correlation of serum l g (asprosin) levels with clinical variables in all subjects.

Variables	Simple		Multiple	
	*r*	*P* value	*β* ± SE	*P* value
Age	0.003	0.961		
BMI	0.206	<0.001		
Waist circumference^#^	0.171	0.003		
Fat (%)	0.113	0.053		
SBP	0.102	0.082		
DBP	0.111	0.058		
TC^a^	−0.022	0.710		
TG^a^	0.251	<0.001	0.033 ± 0.010	0.001
HDL cholesterol	−0.194	0.001		
LDL cholesterol	0.012	0.842		
FPG^a^	0.302	<0.001		
2h-PG^a#^	0.366	<0.001		
FIns^a^	0.314	<0.001		
HOMA-IR^a^	0.316	<0.001	0.028 ± 0.006	<0.001
IL-6	0.183	0.002		
CMP-1	0.175	0.005		

^a^Log-transformed before analysis. ^#^WC did not enter the multivariate regression due to its high intercorrelation with BMI (*r* = 0.746). 2-hour blood glucose did not enter the multivariate regression due to its high intercorrelation with fasting blood glucose (*r* = 0.931). In multiple linear regression analysis, values included for analysis were age, gender, BMI, TG, HDL-C, FPG, FIns, HOMA-IR, IL-6, and MCP-1. BMI, body mass index; Fat, percentage of body fat; SBP, systolic blood pressure; DBP, diastolic blood pressure; TC, total cholesterol; TG, triglyceride, LDL, low-density lipoprotein; HDL, high-density lipoprotein; FPG, fasting plasma glucose; 2h-PG, 2-hour plasma glucose; FIns, fasting insulin; HOMA-IR, homeostasis model assessment of insulin resistance; IL-6, interleukin-6; MCP-1, monocyte chemotactic protein-1.

**Table 3:** Association of serum asprosin with MetS and IR in fully adjusted models.

Model adjustment	MetS			IR		
	*β*	OR per 1 SD increase (95% CI)	*P* value	*β*	OR per 1 SD increase (95% CI)	*P* value
Model 1	0.014	1.076 (1.047, 1.106)	<0.001	0.071	1.074 (1.046, 1.102)	<0.001
Model 2	0.066	1.068 (1.039, 1.745)	<0.001	0.067	1.069 (1.042, 1.097)	<0.001
Model 3	0.055	1.056 (1.022, 1.092)	0.001	0.069	1.071 (1.042, 1.101)	<0.001
Model 4	0.052	1.054 (1.019, 1.089)	0.002	0.067	1.070 (1.041, 1.100)	<0.001
Model 5	0.044	1.044 (1.009, 1.081)	0.014			

Model 1, adjusted for age and gender; Model 2, further adjusted for BMI; Model 3, further adjusted for lipid profiles (TC, TG, LDL-C, and HDL-C); Model 4, further adjusted for inflammatory markers (IL-6 and MCP-1); Model 5, further adjusted for HOMA-IR. IR, insulin resistance; BMI, body mass index; TC, total cholesterol; TG, triglycerides, LDL-C, low-density lipoprotein cholesterol; HDL-C, high-density lipoprotein cholesterol; IL-6, interleukin-6; MCP-1, monocyte chemotactic protein-1. HOMA-IR, homeostasis model assessment of insulin resistance.

**Table 4:** *Row mean scores* and *Cochran–Armitage* trend tests of the impact of asprosin on MetS.

	MetS	
	*χ* ^*2*^	*P* value
*Row mean scores* test	37.248	<0.001
*Cochran–Armitage* test	42.567	<0.001

## Data Availability

The data used to support the findings of this study are available from the corresponding author upon request.

## Supplementary Materials

Supplementary Materials The data of study subjects. The data included anthropometric parameters and biochemical parameters in 162 control subjects and 131 patients of metabolic syndrome.
