# Supplementary material for: High Serum Asprosin Levels Are Associated with Presence of Metabolic Syndrome
Source: Int J Endocrinol. 2021 Mar 2;2021:6622129. doi: 10.1155/2021/6622129 (PMC7943292; doi:10.1155/2021/6622129)
Supplement: Supplementary Materials — The data of study subjects. The data included anthropometric parameters and biochemical parameters in 162 control subjects and 131 patients of metabolic syndrome. [file 6622129.f1.docx]

| MetS | No. of components | Age | Gender | Smoking | High | Weight | BMI | Waist | Fat% | SBP | DBP | TC | TG | LDLC | HDLC | FPG | 2h PG | FINS | HOMA-IR | IL-6 | MCP-1 | Asprosin |
| --- | --- | --- | --- | --- | --- | --- | --- | --- | --- | --- | --- | --- | --- | --- | --- | --- | --- | --- | --- | --- | --- | --- |
| No | 0 | 27 | Female | No | 157 | 62 | 25.44 | 79 | 27.5 | 107 | 83 | 3.96 | 1 | 2.08 | 1.63 | 4.86 | 5.26 | 7 | 1.51 | 8.61 | 105.9 | 9.07 |
| No | 1 | 29 | Female | No | 157 | 53 | 21.52 | 80 | 25.7 | 120 | 69 | 4.72 | 1.36 | 2.42 | 1.68 | 5.02 | 5.71 | 7.21 | 1.61 | 14.6 | 158.27 | 18.04 |
| No | 0 | 30 | Male | No | 162 | 65 | 24.77 | 81 | 27.5 | 111 | 75 | 3.97 | 0.67 | 2.02 | 1.65 | 4.53 | 5.18 | 8.18 | 1.65 | 9.73 | 97.35 | 16.51 |
| No | 1 | 31 | Male | Yes | 164 | 63 | 23.31 | 84 | 27.5 | 118 | 68 | 4.24 | 1.81 | 2.22 | 1.2 | 5.29 | 6.25 | 6.14 | 1.44 | 15.73 | 143.76 | 13.5 |
| No | 0 | 32 | Female | No | 171 | 57 | 19.46 | 72 | 25.4 | 129 | 81 | 4.06 | 0.91 | 1.81 | 1.84 | 4.91 | 5.49 | 5.67 | 1.24 | 10.6 | 82.27 | 6.77 |
| No | 1 | 32 | Female | No | 163 | 68 | 25.75 | 81 | 27.7 | 112 | 68 | 3.87 | 0.89 | 2.15 | 1.32 | 5.39 | 5.82 | 8.43 | 2.02 | 4.83 | 62.32 | 25.44 |
| No | 0 | 33 | Female | No | 154 | 56 | 23.61 | 70 | 19.4 | 114 | 78 | 4.08 | 0.47 | 1.83 | 2.04 | 4.41 | 5.44 | 5.23 | 1.03 | 5.99 | 62.7 | 8.6 |
| No | 0 | 34 | Female | No | 158 | 58 | 23.19 | 79 | 28.8 | 118 | 81 | 4.3 | 0.48 | 1.85 | 2.23 | 5.18 | 5.84 | 6.87 | 1.58 | 19.25 | 153.21 | 10.26 |
| No | 0 | 34 | Female | No | 163 | 56 | 21.04 | 77 | 27.5 | 123 | 75 | 3.79 | 0.43 | 1.84 | 1.75 | 4.92 | 5.75 | 6.91 | 1.51 | 8.67 | 160.93 | 14.09 |
| No | 0 | 34 | Male | Yes | 154 | 59 | 24.67 | 80 | 27.4 | 117 | 68 | 3.5 | 0.32 | 1.91 | 1.44 | 5.11 | 6.04 | 7.59 | 1.72 | 12.24 | 154.68 | 15.2 |
| No | 0 | 34 | Male | Yes | 152 | 44 | 18.83 | 67 | 21.6 | 115 | 67 | 3.53 | 0.74 | 1.79 | 1.4 | 4.67 | 5.7 | 6.75 | 1.4 | 6.2 | 126.67 | 17.4 |
| No | 0 | 34 | Male | Yes | 156 | 52 | 21.41 | 74 | 23.6 | 115 | 75 | 3.89 | 0.81 | 2 | 1.52 | 5.11 | 5.47 | 6.17 | 1.4 | 8.75 | 46.67 | 23.2 |
| No | 1 | 34 | Male | Yes | 167 | 59 | 21.05 | 77 | 26.4 | 137 | 84 | 3.69 | 1.65 | 1.64 | 1.3 | 5.12 | 5.71 | 9.94 | 2.26 | 4.6 | 116.27 | 27.4 |
| No | 2 | 34 | Male | No | 172 | 96 | 32.31 | 102 | 28.5 | 140 | 94 | 5.51 | 1.25 | 3.53 | 1.41 | 4.78 | 5.67 | 8.33 | 1.77 | 21.75 | 205.61 | 13.54 |
| No | 1 | 35 | Male | No | 157 | 50 | 20.41 | 67 | 20.6 | 143 | 92 | 5.68 | 0.56 | 3.57 | 1.86 | 4.04 | 5 | 7.04 | 1.26 | 15.42 | 146.67 | 12.05 |
| No | 1 | 35 | Male | Yes | 172 | 72 | 24.34 | 74 | 24.5 | 109 | 61 | 3.71 | 0.46 | 1.9 | 1.6 | 8.1 | 12.54 | 10.58 | 3.81 | 19.54 | 148.31 | 26.77 |
| No | 1 | 35 | Male | No | 180 | 88 | 27.31 | 96 | 32.5 | 115 | 65 | 4.48 | 1.43 | 2.41 | 1.42 | 5.34 | 6.27 | 6.66 | 1.58 | 18.55 | 135.66 | 15.99 |
| No | 1 | 36 | Male | No | 166 | 65 | 23.59 | 91 | 30.8 | 122 | 74 | 4.7 | 0.55 | 2.73 | 1.73 | 5.09 | 6.06 | 7.23 | 1.64 | 16.63 | 109.04 | 17.4 |
| No | 1 | 37 | Male | No | 157 | 50 | 20.41 | 67 | 21.5 | 163 | 95 | 5.68 | 0.56 | 3.57 | 1.41 | 4.65 | 5.71 | 11.72 | 2.42 | 15.42 | 146.67 | 20.15 |
| No | 0 | 38 | Female | No | 161 | 51 | 19.84 | 77 | 28.4 | 106 | 62 | 5.26 | 1.35 | 2.49 | 1.33 | 4.85 | 5.71 | 6.32 | 1.36 | 8.2 | 98.93 | 17.74 |
| No | 0 | 38 | Female | No | 156 | 49 | 20.01 | 68 | 24.1 | 106 | 59 | 4.11 | 1.12 | 1.76 | 1.84 | 5.09 | 6.04 | 4.76 | 1.08 | 18.2 | 178.93 | 36.96 |
| No | 2 | 38 | Female | No | 152 | 55 | 23.88 | 82 | 28.1 | 131 | 77 | 4.53 | 0.82 | 2.75 | 1.41 | 4.53 | 5.58 | 5.04 | 1.01 | 14.7 | 89.39 | 10.56 |
| No | 1 | 38 | Male | No | 156 | 64 | 26.3 | 86 | 25.8 | 124 | 74 | 2.58 | 0.66 | 1.15 | 1.13 | 5.49 | 5.78 | 6.39 | 1.56 | 8.27 | 53.44 | 22.72 |
| No | 2 | 39 | Female | No | 158 | 57 | 22.67 | 78 | 22.7 | 137 | 85 | 5.25 | 2.25 | 1.91 | 1.32 | 5.31 | 6.11 | 5.5 | 1.3 | 8.26 | 83.01 | 15.06 |
| No | 0 | 39 | Male | Yes | 163 | 60 | 22.43 | 74 | 25.1 | 110 | 61 | 4.48 | 0.63 | 1.57 | 1.51 | 4.71 | 5.37 | 8.05 | 1.69 | 13.62 | 110.11 | 17.4 |
| No | 1 | 39 | Male | No | 151 | 42 | 18.5 | 61 | 25.8 | 113 | 74 | 3.69 | 0.93 | 1.55 | 1.29 | 13.31 | 19.4 | 10.43 | 6.17 | 9.8 | 123.6 | 24.84 |
| No | 2 | 39 | Female | No | 157 | 59 | 23.94 | 85 | 29.6 | 100 | 72 | 4.96 | 1.35 | 1.35 | 1.23 | 4.95 | 5.71 | 7.18 | 1.58 | 21.8 | 145.68 | 14.28 |
| No | 1 | 39 | Female | No | 158 | 60 | 23.95 | 81 | 20.7 | 105 | 66 | 3.63 | 0.94 | 1.67 | 1.53 | 4.63 | 4.81 | 5.25 | 1.08 | 8.73 | 100.22 | 24.84 |
| No | 1 | 41 | Female | No | 162 | 75 | 28.58 | 75 | 25.4 | 133 | 68 | 5.03 | 0.57 | 2.97 | 1.8 | 5.11 | 5.94 | 3.76 | 0.85 | 18.02 | 138 | 11.71 |
| No | 0 | 41 | Male | Yes | 158 | 50 | 19.95 | 66 | 23.7 | 110 | 65 | 4.11 | 0.82 | 1.58 | 2.16 | 4.82 | 5.7 | 6.02 | 1.29 | 15.83 | 195.33 | 20.45 |
| No | 2 | 41 | Female | No | 151 | 57 | 25.08 | 85 | 29.6 | 99 | 72 | 3.75 | 1.03 | 2.17 | 1.11 | 4.93 | 5.63 | 6.49 | 1.42 | 14.71 | 102.29 | 25.81 |
| No | 0 | 42 | Male | No | 162 | 61 | 23.39 | 73 | 26.5 | 91 | 58 | 5.12 | 1.37 | 1.71 | 1.79 | 4.92 | 5.81 | 7.29 | 1.59 | 19.09 | 108.24 | 17.22 |
| No | 1 | 42 | Male | No | 156 | 52 | 21.46 | 84 | 28.5 | 137 | 81 | 4.82 | 1.47 | 2.1 | 1.55 | 5.52 | 6.24 | 5.52 | 1.35 | 3 | 94.27 | 21.64 |
| No | 1 | 42 | Female | No | 158 | 51 | 20.36 | 81 | 25.4 | 113 | 66 | 3.93 | 0.95 | 1.81 | 1.69 | 4.21 | 5.2 | 5.32 | 1 | 15.42 | 126.67 | 13.54 |
| No | 2 | 42 | Male | Yes | 180 | 73 | 22.59 | 92 | 31.2 | 121 | 82 | 5.37 | 2.21 | 2.48 | 1.18 | 5.23 | 6.11 | 5.29 | 1.23 | 22.61 | 133.4 | 16.51 |
| No | 0 | 43 | Male | No | 157 | 56 | 22.72 | 73 | 23.4 | 125 | 75 | 4.93 | 1.16 | 2.78 | 1.56 | 5.24 | 6.71 | 6.24 | 1.45 | 7.07 | 114.75 | 8.26 |
| No | 2 | 43 | Female | No | 156 | 54 | 22.35 | 78 | 21.7 | 132 | 68 | 4.26 | 0.87 | 2.37 | 1.49 | 11.57 | 17.34 | 5.6 | 2.88 | 10.83 | 114.87 | 9.59 |
| No | 2 | 43 | Male | No | 164 | 64 | 23.8 | 81 | 27 | 138 | 84 | 4.73 | 1.49 | 2.57 | 1.53 | 8.76 | 12.58 | 11.34 | 4.42 | 11.2 | 110.27 | 9.97 |
| No | 1 | 43 | Male | Yes | 168 | 68 | 24.09 | 84 | 25.1 | 125 | 80 | 4.63 | 1.41 | 1.89 | 1.08 | 7.25 | 15.47 | 13.21 | 4.26 | 25.08 | 130.04 | 25.44 |
| No | 2 | 43 | Female | No | 152 | 49 | 21.38 | 80 | 27.1 | 138 | 79 | 5.97 | 1.3 | 3.29 | 2.09 | 5.37 | 6.27 | 7.34 | 1.75 | 9.36 | 201.33 | 13.13 |
| No | 2 | 43 | Female | No | 160 | 70 | 27.34 | 98 | 31.6 | 150 | 80 | 3.39 | 1.67 | 1.6 | 1.43 | 5.16 | 5.94 | 6.32 | 1.45 | 11.54 | 125.66 | 13.5 |
| No | 1 | 44 | Male | No | 170 | 69 | 23.84 | 77 | 27.5 | 129 | 80 | 3.62 | 1.42 | 2.02 | 0.95 | 4.83 | 5.29 | 7.78 | 1.67 | 15.44 | 121.05 | 10.78 |
| No | 2 | 44 | Female | No | 146 | 46 | 21.53 | 82 | 27.1 | 132 | 81 | 3.26 | 0.53 | 1.42 | 1.5 | 5.1 | 6.14 | 8.92 | 2.02 | 6.2 | 152.27 | 15.25 |
| No | 1 | 44 | Female | No | 164 | 57 | 21.16 | 88 | 29.8 | 116 | 61 | 3.73 | 0.8 | 1.43 | 1.94 | 4.55 | 4.75 | 4.72 | 0.95 | 10 | 98.67 | 15.58 |
| No | 1 | 45 | Female | No | 162 | 59 | 22.48 | 78 | 26.4 | 123 | 70 | 5.59 | 0.76 | 3.32 | 1.92 | 5.88 | 6.77 | 9.88 | 2.58 | 19.39 | 123.53 | 14.21 |
| No | 2 | 45 | Male | No | 163 | 67 | 25.22 | 79 | 24.1 | 123 | 64 | 6.1 | 6.14 | 1.89 | 1.42 | 15.15 | 20.68 | 13.93 | 9.38 | 13.81 | 124.04 | 21.12 |
| No | 2 | 45 | Female | No | 152 | 60 | 25.97 | 87 | 29.3 | 131 | 77 | 5.26 | 1.51 | 2.96 | 1.61 | 5.46 | 5.81 | 7.94 | 1.93 | 7.62 | 237.58 | 18.82 |
| No | 2 | 45 | Female | No | 153 | 73 | 31.18 | 102 | 32.7 | 124 | 79 | 3.36 | 0.89 | 2.54 | 1.37 | 8.91 | 17.37 | 10.43 | 4.13 | 6.6 | 34.93 | 39.53 |
| No | 0 | 46 | Male | No | 161 | 53 | 20.42 | 75 | 21.9 | 128 | 62 | 4.35 | 0.7 | 2.74 | 1.29 | 4.46 | 5.67 | 6.1 | 1.21 | 7.8 | 119.6 | 13.28 |
| No | 1 | 46 | Female | No | 148 | 48 | 21.87 | 74 | 27.6 | 115 | 73 | 4.18 | 0.62 | 2.08 | 1.27 | 4.85 | 5.81 | 5.37 | 1.16 | 13.08 | 127.36 | 20.86 |
| No | 1 | 46 | Female | No | 151 | 54 | 23.8 | 75 | 14.5 | 117 | 78 | 5.69 | 3.35 | 2.42 | 1.75 | 4.56 | 5.58 | 6.66 | 1.35 | 8.39 | 74.28 | 28.04 |
| No | 2 | 46 | Female | No | 161 | 63 | 24.38 | 93 | 37.5 | 135 | 76 | 4.09 | 0.86 | 2.28 | 1.42 | 5.44 | 6.02 | 8.85 | 2.14 | 8.37 | 93.24 | 13.7 |
| No | 2 | 46 | Male | Yes | 165 | 60 | 22.25 | 85 | 27.5 | 135 | 70 | 5.34 | 1.25 | 2.54 | 1.17 | 5.07 | 6.11 | 5.6 | 1.26 | 15.36 | 68.44 | 15.8 |
| No | 0 | 47 | Male | No | 161 | 61 | 23.34 | 81 | 24.1 | 101 | 62 | 4.73 | 1.04 | 2.61 | 1.65 | 5.22 | 6.17 | 4.34 | 1.01 | 8.56 | 69.23 | 9.07 |
| No | 1 | 47 | Female | No | 154 | 52 | 22.11 | 79 | 27.6 | 127 | 74 | 5.88 | 1.25 | 3.66 | 1.65 | 5.61 | 6.52 | 4.82 | 1.2 | 5.72 | 80.06 | 9.15 |
| No | 0 | 47 | Female | No | 160 | 51 | 19.84 | 72 | 25.4 | 117 | 70 | 3.73 | 0.66 | 2.08 | 1.35 | 4.43 | 5.19 | 6.35 | 1.25 | 5.8 | 65.6 | 17.66 |
| No | 1 | 47 | Male | Yes | 165 | 67 | 24.61 | 72 | 23.7 | 121 | 73 | 5.02 | 1.35 | 3.7 | 0.95 | 4.69 | 5.38 | 7.16 | 1.49 | 7.28 | 76.91 | 19.6 |
| No | 2 | 47 | Female | No | 146 | 51 | 23.9 | 81 | 26.4 | 118 | 67 | 8.83 | 1.14 | 6.43 | 1.88 | 5.07 | 5.97 | 6.09 | 1.37 | 7.08 | 56 | 16.03 |
| No | 2 | 47 | Female | No | 169 | 71 | 24.72 | 84 | 27.5 | 106 | 65 | 4.23 | 1.25 | 2.39 | 1.27 | 4.63 | 4.92 | 8.92 | 1.84 | 10.87 | 113.04 | 36.52 |
| No | 0 | 48 | Female | No | 161 | 56 | 21.64 | 77 | 24.6 | 124 | 79 | 6.52 | 0.49 | 2.71 | 3.59 | 4.93 | 5.66 | 6.24 | 1.37 | 15.03 | 110.21 | 8.66 |
| No | 0 | 48 | Male | No | 163 | 62 | 23.37 | 79 | 26.8 | 123 | 79 | 4.99 | 1.39 | 1.78 | 1.58 | 4.7 | 5.71 | 5.83 | 1.22 | 11.82 | 84.36 | 9 |
| No | 0 | 48 | Female | No | 165 | 60 | 22.04 | 78 | 26.8 | 125 | 79 | 3.61 | 0.84 | 1.6 | 1.63 | 4.95 | 5.33 | 7.04 | 1.55 | 9.03 | 68.41 | 12.72 |
| No | 2 | 48 | Male | No | 154 | 56 | 23.78 | 82 | 26.1 | 134 | 76 | 7.84 | 0.68 | 5.97 | 1.56 | 10.81 | 18.48 | 15 | 7.21 | 23.62 | 163.45 | 13.35 |
| No | 0 | 48 | Male | No | 161 | 59 | 22.76 | 79 | 23.8 | 126 | 75 | 5.56 | 0.64 | 3.98 | 1.29 | 4.72 | 5.34 | 7.71 | 1.62 | 10.7 | 119.96 | 14.13 |
| No | 1 | 48 | Male | No | 167 | 66 | 23.77 | 82 | 27.1 | 131 | 74 | 4.01 | 1.59 | 1.99 | 1.3 | 4.82 | 5.49 | 9.74 | 2.09 | 19.81 | 143.65 | 16.7 |
| No | 1 | 48 | Male | No | 179 | 76 | 23.63 | 79 | 26.8 | 137 | 79 | 3.31 | 0.91 | 1.58 | 1.32 | 4.73 | 4.92 | 5.66 | 1.19 | 11.66 | 123.69 | 17.9 |
| No | 2 | 48 | Male | No | 170 | 82 | 28.37 | 80 | 31.2 | 132 | 73 | 5.46 | 0.99 | 3.21 | 1.8 | 9.8 | 13.69 | 12.85 | 5.6 | 5 | 58.93 | 23.2 |
| No | 0 | 48 | Female | No | 155 | 53 | 21.94 | 78 | 24.7 | 127 | 77 | 4.28 | 1.2 | 1.61 | 1.6 | 4.96 | 5.74 | 6.85 | 1.51 | 16.8 | 125.18 | 25.77 |
| No | 2 | 48 | Female | No | 149 | 53 | 23.94 | 87 | 30.4 | 135 | 81 | 5.19 | 1.24 | 2.99 | 1.64 | 5.44 | 6.33 | 8.64 | 2.09 | 6.55 | 61.02 | 15.43 |
| No | 2 | 48 | Female | No | 159 | 59 | 23.14 | 81 | 27.8 | 121 | 70 | 5.17 | 1.63 | 2.36 | 1.01 | 5.52 | 6.04 | 9.51 | 2.33 | 5.36 | 73.98 | 26.8 |
| No | 2 | 48 | Female | No | 154 | 55 | 23.19 | 86 | 27.5 | 120 | 80 | 3.88 | 1.13 | 1.99 | 1.38 | 12.26 | 19.66 | 11.81 | 6.44 | 19.35 | 143.08 | 28.45 |
| No | 0 | 49 | Male | Yes | 163 | 59 | 22.23 | 79 | 32.2 | 124 | 78 | 3.86 | 1.05 | 2.08 | 1.3 | 4.55 | 5.49 | 7.25 | 1.47 | 7.22 | 46.33 | 9.22 |
| No | 0 | 49 | Male | Yes | 162 | 62 | 23.62 | 76 | 22.6 | 122 | 75 | 4.84 | 1.61 | 2.53 | 1.58 | 5.02 | 6.16 | 9.73 | 2.17 | 8.26 | 66.46 | 15.1 |
| No | 1 | 49 | Female | No | 159 | 56 | 22.21 | 84 | 28.2 | 118 | 71 | 4.61 | 0.77 | 1.8 | 2.46 | 5.46 | 6.02 | 3.39 | 0.82 | 17.35 | 145.69 | 7.2 |
| No | 1 | 49 | Female | No | 149 | 52 | 23.38 | 84 | 28.5 | 127 | 73 | 4.94 | 0.52 | 2.5 | 2.2 | 4.85 | 5.26 | 5.37 | 1.16 | 9.73 | 77.43 | 8.66 |
| No | 1 | 49 | Male | Yes | 167 | 62 | 22.44 | 88 | 29.8 | 109 | 75 | 5.51 | 0.94 | 3.45 | 1.63 | 4.6 | 5.48 | 8.21 | 1.68 | 18.26 | 158.33 | 11.3 |
| No | 1 | 49 | Female | No | 170 | 64 | 22.35 | 82 | 26.3 | 106 | 72 | 3.99 | 0.72 | 2.32 | 1.34 | 5.34 | 6.18 | 13.54 | 3.21 | 18.01 | 172.41 | 20.6 |
| No | 1 | 50 | Female | No | 152 | 45 | 19.56 | 71 | 27.5 | 149 | 95 | 5.11 | 1.52 | 2.45 | 1.97 | 4.62 | 5.27 | 4.71 | 0.97 | 13 | 109.6 | 9.85 |
| No | 0 | 50 | Male | Yes | 170 | 74 | 25.61 | 82 | 33.6 | 112 | 69 | 4.64 | 0.73 | 3.02 | 1.29 | 4.2 | 5.16 | 8.96 | 1.67 | 4.97 | 93.05 | 11.27 |
| No | 1 | 50 | Male | Yes | 163 | 56 | 21.02 | 71 | 26.4 | 148 | 85 | 5.85 | 0.83 | 3.9 | 1.57 | 5.11 | 5.94 | 10.39 | 2.36 | 13 | 109.6 | 13.72 |
| No | 1 | 50 | Female | No | 152 | 50 | 21.81 | 76 | 25.8 | 102 | 74 | 3.18 | 0.69 | 1.83 | 1.04 | 5.1 | 6.07 | 7.23 | 1.64 | 19.72 | 120.93 | 15.06 |
| No | 1 | 50 | Female | No | 157 | 52 | 21.18 | 75 | 24.9 | 114 | 70 | 5.2 | 1.49 | 3.22 | 1.3 | 4.8 | 4.92 | 8.33 | 1.78 | 12.2 | 98.27 | 15.36 |
| No | 1 | 50 | Male | No | 168 | 68 | 24.24 | 78 | 27.5 | 103 | 58 | 3.35 | 1.81 | 1.51 | 1.02 | 4.98 | 5.68 | 6.24 | 1.38 | 15.73 | 149.68 | 15.47 |
| No | 0 | 50 | Female | No | 159 | 55 | 21.6 | 77 | 28.6 | 127 | 82 | 4.67 | 1.24 | 1.02 | 2 | 4.44 | 5.63 | 6.07 | 1.2 | 9.58 | 66 | 21.72 |
| No | 0 | 50 | Female | No | 162 | 62 | 23.43 | 79 | 25.1 | 113 | 67 | 5.2 | 0.75 | 2.75 | 2.11 | 4.53 | 5.68 | 8.76 | 1.76 | 15.75 | 95.04 | 31.83 |
| No | 1 | 50 | Female | No | 152 | 52 | 22.46 | 71 | 27.4 | 153 | 80 | 5.58 | 1.64 | 3.12 | 1.71 | 4.54 | 4.72 | 9.04 | 1.82 | 16.05 | 134.42 | 35.4 |
| No | 2 | 50 | Male | Yes | 157 | 55 | 22.39 | 79 | 28.8 | 149 | 88 | 3.75 | 1.01 | 2.05 | 1.24 | 7.72 | 16.71 | 10.62 | 3.64 | 8.04 | 94.27 | 53.54 |
| No | 1 | 50 | Male | Yes | 169 | 68 | 23.77 | 85 | 26.3 | 109 | 61 | 3.85 | 0.87 | 1.64 | 1.88 | 5.36 | 5.37 | 6.71 | 1.6 | 12.73 | 103.57 | 5.32 |
| No | 2 | 50 | Male | No | 169 | 68 | 23.77 | 85 | 26.2 | 109 | 61 | 4.75 | 1.39 | 2.69 | 1.58 | 6.56 | 7.39 | 8.62 | 2.51 | 11.8 | 84.3 | 16.6 |
| No | 2 | 50 | Male | No | 165 | 73 | 26.89 | 93 | 31.6 | 119 | 80 | 6.46 | 3.33 | 2.9 | 1.05 | 5.05 | 5.72 | 8.46 | 1.9 | 15.72 | 203.62 | 22.7 |
| No | 0 | 51 | Male | No | 156 | 55 | 22.75 | 79 | 29.2 | 124 | 71 | 4.72 | 0.8 | 2.67 | 1.69 | 4.74 | 5.34 | 9.1 | 1.92 | 9.03 | 97.42 | 16.77 |
| No | 0 | 51 | Male | No | 167 | 62 | 22.51 | 84 | 28.5 | 104 | 64 | 4.53 | 1.19 | 2.4 | 1.59 | 4.61 | 5.38 | 4.83 | 0.99 | 7.71 | 64.25 | 17.11 |
| No | 1 | 51 | Male | Yes | 162 | 57 | 21.72 | 80 | 29.3 | 131 | 81 | 4.92 | 1.21 | 2.45 | 1.6 | 5.23 | 5.61 | 8.46 | 1.97 | 10.21 | 84.2 | 20.01 |
| No | 2 | 51 | Female | No | 150 | 49 | 21.73 | 77 | 26.1 | 134 | 86 | 3.02 | 1.13 | 0.73 | 1.46 | 11.57 | 17.34 | 5.6 | 2.88 | 10.92 | 107.31 | 23.54 |
| No | 1 | 51 | Male | No | 163 | 65 | 24.46 | 90 | 28.8 | 121 | 84 | 5.53 | 0.96 | 3.65 | 1.84 | 4.82 | 5.93 | 6.24 | 1.34 | 6.29 | 84.68 | 7.66 |
| No | 1 | 51 | Female | No | 146 | 50 | 23.41 | 86 | 26.5 | 122 | 74 | 4.83 | 0.72 | 2.3 | 2.2 | 5.21 | 6.38 | 3.57 | 0.83 | 10.24 | 76.6 | 10.4 |
| No | 2 | 51 | Female | No | 158 | 59 | 23.63 | 85 | 24.6 | 151 | 85 | 4.61 | 1.31 | 2.19 | 1.82 | 5.13 | 5.74 | 9.08 | 2.07 | 17.85 | 96.8 | 19.5 |
| No | 2 | 51 | Male | No | 171 | 71 | 24.49 | 90 | 31.4 | 116 | 77 | 5.04 | 1.11 | 3.19 | 1.35 | 9.8 | 13.69 | 12.85 | 5.6 | 18 | 105.6 | 21.75 |
| No | 2 | 52 | Male | No | 175 | 72 | 23.51 | 80 | 27.8 | 130 | 81 | 3.53 | 1.95 | 1.49 | 1.42 | 5.24 | 5.86 | 4.97 | 1.16 | 14.6 | 117.02 | 15.43 |
| No | 1 | 52 | Female | No | 163 | 68 | 25.56 | 75 | 26.8 | 113 | 67 | 4.68 | 1.83 | 2.07 | 1.58 | 5.26 | 5.74 | 6.66 | 1.56 | 16.69 | 132.02 | 24.8 |
| No | 0 | 52 | Female | No | 149 | 44 | 20 | 67 | 24.5 | 119 | 73 | 6.74 | 1.36 | 4.42 | 1.81 | 4.36 | 5.2 | 8.77 | 1.7 | 13.4 | 148.87 | 47.3 |
| No | 2 | 52 | Female | No | 164 | 69 | 25.65 | 88 | 29.8 | 126 | 62 | 5.21 | 2.15 | 2.18 | 1.56 | 5.11 | 5.84 | 4.38 | 0.99 | 13.74 | 116.08 | 19.67 |
| No | 0 | 53 | Male | No | 176 | 66 | 21.4 | 75 | 25.4 | 90 | 60 | 3.46 | 0.92 | 1.19 | 1.23 | 5.47 | 5.77 | 8.47 | 2.06 | 11 | 163.6 | 12.87 |
| No | 2 | 53 | Male | No | 165 | 62 | 22.77 | 77 | 27.8 | 135 | 78 | 6.46 | 1.23 | 4.37 | 1.53 | 8.42 | 12.52 | 11.29 | 4.22 | 10.82 | 139.04 | 13.24 |
| No | 2 | 53 | Male | No | 161 | 61 | 23.42 | 83 | 28.9 | 125 | 88 | 3.12 | 1.2 | 1.23 | 1.34 | 10.66 | 18.07 | 8.3 | 3.93 | 14.95 | 121.75 | 19.41 |
| No | 0 | 53 | Male | No | 162 | 67 | 25.53 | 83 | 27.5 | 127 | 76 | 5.03 | 0.49 | 2.76 | 2.01 | 4.73 | 5.17 | 6.56 | 1.38 | 18.28 | 137.76 | 24.69 |
| No | 2 | 53 | Female | No | 160 | 60 | 23.32 | 81 | 23.9 | 131 | 83 | 5.95 | 1.58 | 3.85 | 1.38 | 5.5 | 6.43 | 7.55 | 1.85 | 18.07 | 134.55 | 24.21 |
| No | 0 | 54 | Male | Yes | 163 | 57 | 21.45 | 79 | 26.4 | 102 | 68 | 5.13 | 1.48 | 3.02 | 1.44 | 4.46 | 5.48 | 5.61 | 1.11 | 13.75 | 134 | 12.87 |
| No | 1 | 54 | Female | No | 151 | 51 | 22.15 | 76 | 32.5 | 138 | 85 | 7.47 | 1.35 | 5.15 | 1.71 | 5.15 | 6.06 | 6.82 | 1.56 | 11.77 | 112.49 | 15.88 |
| No | 1 | 54 | Female | No | 167 | 56 | 20.08 | 87 | 31.7 | 101 | 79 | 4.44 | 0.67 | 2.29 | 1.85 | 4.69 | 5.62 | 5.37 | 1.12 | 13.8 | 96.93 | 15.84 |
| No | 1 | 54 | Male | No | 157 | 64 | 26.05 | 86 | 24.4 | 96 | 59 | 4.26 | 1.08 | 2.3 | 1.48 | 4.63 | 5.39 | 7.19 | 1.48 | 11.35 | 138.37 | 17.66 |
| No | 0 | 55 | Male | No | 167 | 64 | 22.98 | 73 | 27.8 | 128 | 87 | 4.66 | 0.9 | 2.89 | 1.36 | 4.96 | 6.03 | 7.44 | 1.64 | 9.82 | 98.26 | 16.03 |
| No | 1 | 55 | Male | Yes | 174 | 66 | 21.73 | 75 | 25.3 | 132 | 81 | 5.18 | 1.45 | 2.81 | 1.71 | 4.96 | 5.96 | 7.19 | 1.58 | 14.73 | 188.76 | 17.7 |
| No | 0 | 55 | Female | No | 165 | 54 | 19.99 | 78 | 29.6 | 87 | 63 | 4.14 | 1.47 | 1.78 | 1.69 | 5.1 | 5.9 | 8.27 | 1.87 | 7.4 | 115.6 | 18.74 |
| No | 2 | 55 | Male | Yes | 163 | 60 | 22.58 | 77 | 85.4 | 137 | 85 | 5.67 | 2.46 | 2.31 | 1.33 | 5.12 | 5.9 | 7.96 | 1.81 | 8.28 | 93.42 | 19.2 |
| No | 0 | 55 | Female | No | 156 | 50 | 20.63 | 75 | 26.3 | 128 | 71 | 4.65 | 1.26 | 1.62 | 1.33 | 4.37 | 4.82 | 7.92 | 1.54 | 10 | 40.67 | 20.3 |
| No | 1 | 55 | Female | No | 152 | 45 | 19.56 | 71 | 27.4 | 132 | 84 | 5.11 | 1.52 | 2.45 | 1.97 | 5.42 | 5.92 | 6.9 | 1.66 | 4.6 | 192.27 | 20.75 |
| No | 1 | 55 | Male | Yes | 169 | 58 | 20.57 | 82 | 28.6 | 129 | 75 | 5.26 | 2.41 | 1.51 | 1.13 | 5.18 | 6.27 | 7.94 | 1.83 | 9.58 | 160.67 | 23.13 |
| No | 2 | 55 | Male | No | 177 | 72 | 22.89 | 86 | 23.1 | 122 | 68 | 4.68 | 0.97 | 2.41 | 1.83 | 14.64 | 24.52 | 11.29 | 7.34 | 9.93 | 164.67 | 11.71 |
| No | 1 | 55 | Female | No | 161 | 51 | 19.6 | 84 | 27.8 | 127 | 66 | 3.46 | 0.88 | 1.68 | 1.38 | 4.96 | 5.81 | 6.45 | 1.42 | 3.8 | 134.18 | 15.84 |
| No | 1 | 55 | Male | No | 155 | 61 | 25.55 | 93 | 36.4 | 121 | 79 | 5.26 | 1.37 | 3.16 | 1.44 | 5.53 | 6.33 | 10.54 | 2.59 | 22.74 | 182.91 | 18.63 |
| No | 2 | 56 | Male | Yes | 165 | 56 | 20.39 | 81 | 28.8 | 108 | 69 | 3.4 | 0.75 | 1.1 | 0.99 | 7.83 | 8.64 | 10.67 | 3.71 | 17.5 | 158.67 | 14.17 |
| No | 1 | 56 | Female | No | 145 | 48 | 22.83 | 82 | 27.8 | 92 | 54 | 4 | 0.64 | 2.03 | 1.68 | 5.05 | 6.06 | 5.1 | 1.14 | 8.52 | 74.31 | 12.79 |
| No | 1 | 56 | Male | No | 148 | 59 | 27.12 | 89 | 30.2 | 122 | 80 | 5.07 | 1.1 | 2.68 | 1.89 | 4.26 | 5.15 | 7.23 | 1.37 | 10.07 | 97.45 | 16.7 |
| No | 1 | 57 | Female | No | 155 | 44 | 18.52 | 73 | 26.6 | 133 | 84 | 4.12 | 1.01 | 2.22 | 1.44 | 5.12 | 5.29 | 6.94 | 1.58 | 7.08 | 121.33 | 7.03 |
| No | 2 | 57 | Male | No | 157 | 57 | 23.27 | 74 | 23.7 | 152 | 94 | 5.04 | 1.21 | 1.88 | 2.45 | 5.64 | 6.39 | 9.12 | 2.29 | 20.05 | 173.3 | 9.82 |
| No | 1 | 57 | Male | Yes | 156 | 55 | 22.91 | 82 | 28.8 | 112 | 64 | 3.88 | 1.1 | 2.41 | 0.97 | 4.82 | 5.71 | 5.2 | 1.11 | 7.25 | 84.22 | 16.18 |
| No | 0 | 57 | Female | No | 160 | 57 | 22.07 | 77 | 24.4 | 116 | 65 | 3.91 | 0.63 | 2.12 | 1.5 | 4.93 | 5.78 | 5.06 | 1.11 | 33.19 | 214.81 | 22.27 |
| No | 2 | 57 | Male | No | 165 | 65 | 23.95 | 85 | 28.8 | 124 | 69 | 5.33 | 2.24 | 1.69 | 1.36 | 4.51 | 5.32 | 5.7 | 1.14 | 14.57 | 115.33 | 12.53 |
| No | 2 | 57 | Female | No | 162 | 72 | 27.43 | 99 | 30.6 | 120 | 70 | 4.83 | 1.44 | 2.79 | 1.39 | 12.99 | 19.51 | 9.74 | 5.62 | 20.75 | 184.22 | 25.3 |
| No | 2 | 58 | Male | No | 159 | 59 | 23.34 | 82 | 27.4 | 134 | 89 | 5.26 | 2.87 | 2.29 | 1.25 | 4.98 | 5.82 | 7.16 | 1.58 | 9.2 | 202.93 | 26.7 |
| No | 2 | 58 | Female | No | 146 | 49 | 22.85 | 74 | 26.7 | 134 | 77 | 5.44 | 2.12 | 2.91 | 2.02 | 5.02 | 6.07 | 6.07 | 1.35 | 10.07 | 116.24 | 28.56 |
| No | 1 | 58 | Female | No | 160 | 59 | 22.85 | 89 | 26.8 | 114 | 75 | 4.77 | 0.89 | 2.78 | 1.59 | 5.15 | 6.04 | 14.52 | 3.32 | 18.09 | 144.93 | 18.6 |
| No | 2 | 58 | Male | No | 162 | 72 | 27.43 | 93 | 28.4 | 155 | 95 | 4.53 | 1.36 | 1.66 | 1.22 | 4.58 | 5.68 | 7.16 | 1.46 | 17.3 | 157.22 | 45.4 |
| No | 2 | 59 | Female | No | 152 | 57 | 24.63 | 91 | 32.6 | 149 | 92 | 5.42 | 1.02 | 3.5 | 1.66 | 5.16 | 6.22 | 8.21 | 1.88 | 16.76 | 122.04 | 10.26 |
| No | 2 | 59 | Female | No | 154 | 52 | 21.9 | 84 | 21.7 | 131 | 76 | 6.5 | 1.34 | 4.21 | 1.55 | 5.26 | 15.35 | 8.86 | 2.07 | 8.08 | 84.34 | 18.6 |
| No | 2 | 60 | Female | No | 164 | 57 | 21.08 | 85 | 24.7 | 131 | 83 | 4.03 | 0.47 | 1.82 | 2.1 | 4.9 | 6.07 | 5.26 | 1.15 | 15.04 | 144.93 | 16.25 |
| No | 2 | 60 | Female | No | 152 | 66 | 28.76 | 92 | 30.1 | 112 | 66 | 4.85 | 1.32 | 2.69 | 1.17 | 4.39 | 5.28 | 6.83 | 1.33 | 14.74 | 115.93 | 16.7 |
| No | 0 | 60 | Male | Yes | 163 | 87 | 32.74 | 101 | 37.2 | 139 | 87 | 4.79 | 1.38 | 3.39 | 1.11 | 4.93 | 5.86 | 4.63 | 1.01 | 26.63 | 118.27 | 31.83 |
| No | 1 | 60 | Female | No | 158 | 62 | 24.88 | 81 | 28.4 | 111 | 69 | 4.53 | 1.07 | 1.81 | 1.55 | 5.21 | 5.97 | 7.44 | 1.72 | 17.44 | 188.72 | 31.9 |
| No | 2 | 60 | Male | No | 161 | 66 | 25.5 | 99 | 25.4 | 133 | 84 | 5.26 | 1.12 | 3.22 | 1.53 | 4.46 | 5.49 | 5.83 | 1.16 | 17.4 | 195.31 | 38.41 |
| No | 2 | 60 | Male | No | 168 | 63 | 22.32 | 85 | 25.4 | 120 | 71 | 4.95 | 1.04 | 2.75 | 1.53 | 8.89 | 16.38 | 11.43 | 4.52 | 12.08 | 108.47 | 40.6 |
| No | 0 | 61 | Female | No | 157 | 49 | 19.8 | 70 | 26.4 | 119 | 68 | 3.96 | 0.58 | 1.56 | 2.14 | 4.93 | 6.07 | 10.7 | 2.34 | 7.4 | 82.27 | 12.79 |
| No | 2 | 61 | Male | Yes | 177 | 68 | 21.8 | 85 | 30.3 | 138 | 87 | 3.76 | 1.08 | 1.98 | 1.29 | 5.04 | 5.82 | 5.1 | 1.14 | 10.11 | 147.03 | 25.36 |
| No | 2 | 62 | Female | No | 152 | 50 | 21.47 | 78 | 27.8 | 126 | 76 | 4.16 | 1.12 | 1.57 | 1.11 | 14.5 | 20.99 | 11.67 | 7.52 | 7.5 | 104.15 | 12.31 |
| No | 1 | 62 | Female | No | 156 | 54 | 22.17 | 77 | 26.2 | 102 | 60 | 3.9 | 0.99 | 1.53 | 1.92 | 5.67 | 6.38 | 7.71 | 1.94 | 13.83 | 85.04 | 18.93 |
| No | 2 | 62 | Female | No | 163 | 61 | 23.03 | 86 | 27.5 | 138 | 82 | 5.01 | 1.52 | 2.73 | 1.59 | 5.24 | 5.38 | 7.08 | 1.65 | 22 | 124.93 | 39.57 |
| No | 2 | 63 | Female | No | 160 | 65 | 25.35 | 79 | 25.4 | 143 | 83 | 7.52 | 0.98 | 4.75 | 2.32 | 5.66 | 6.53 | 4.66 | 1.17 | 7.33 | 82.54 | 24.2 |
| No | 2 | 63 | Female | No | 153 | 62 | 26.75 | 93 | 24.1 | 131 | 86 | 4.89 | 1.22 | 1.98 | 1.59 | 4.34 | 5.22 | 8.29 | 1.6 | 11.6 | 174.52 | 12.79 |
| No | 2 | 63 | Female | No | 162 | 72 | 27.43 | 96 | 33.8 | 110 | 70 | 4.71 | 1.52 | 1.14 | 1.88 | 13.89 | 17.14 | 10.14 | 6.26 | 14.63 | 163.55 | 31.7 |
| No | 0 | 64 | Male | No | 167 | 58 | 20.76 | 80 | 29.5 | 115 | 72 | 4.13 | 0.85 | 2.46 | 1.25 | 4.52 | 5.32 | 5.04 | 1.01 | 5 | 70.93 | 16.03 |
| No | 1 | 64 | Female | No | 153 | 53 | 22.6 | 75 | 29.5 | 143 | 68 | 5.03 | 0.57 | 2.97 | 1.8 | 5.15 | 5.61 | 7.94 | 1.82 | 9.58 | 128.15 | 18.82 |
| No | 1 | 64 | Female | No | 152 | 61 | 26.27 | 89 | 30.3 | 115 | 53 | 6.74 | 1.04 | 1.45 | 1.77 | 5.19 | 5.81 | 6.91 | 1.59 | 15.05 | 86.93 | 19.41 |
| No | 2 | 65 | Male | No | 165 | 56 | 20.64 | 76 | 25.8 | 132 | 78 | 6.35 | 2.62 | 1.28 | 1.25 | 5.47 | 6.38 | 9.04 | 2.2 | 6.6 | 56 | 21.16 |
| No | 2 | 65 | Female | Yes | 157 | 66 | 26.95 | 92 | 23.7 | 161 | 81 | 4.85 | 1.13 | 2.55 | 1.79 | 5.41 | 5.93 | 7.59 | 1.82 | 10.14 | 146.87 | 6.02 |
| No | 2 | 65 | Female | No | 161 | 69 | 26.79 | 89 | 29.3 | 136 | 78 | 4.29 | 0.82 | 2.18 | 1.72 | 4.87 | 5.67 | 6.52 | 1.41 | 16.84 | 130.57 | 7.3 |
| No | 2 | 65 | Female | No | 170 | 65 | 22.66 | 85 | 29.5 | 133 | 73 | 4.96 | 0.93 | 2.36 | 2.18 | 5.13 | 5.67 | 7.93 | 1.81 | 14.83 | 105.37 | 10.34 |
| No | 2 | 65 | Female | No | 154 | 68 | 28.59 | 95 | 32.7 | 137 | 81 | 5.9 | 1.63 | 3.82 | 1.84 | 5.15 | 6.04 | 7.34 | 1.68 | 16.75 | 184.88 | 24.6 |
| No | 0 | 66 | Male | Yes | 170 | 66 | 22.84 | 82 | 27.4 | 121 | 73 | 3.97 | 0.92 | 1.71 | 1.84 | 4.48 | 4.91 | 7.92 | 1.58 | 13.57 | 102.71 | 12.12 |
| No | 1 | 66 | Female | No | 150 | 52 | 23.11 | 82 | 30.2 | 122 | 80 | 5.04 | 0.99 | 2.38 | 2.21 | 5.26 | 5.93 | 5.1 | 1.19 | 5.82 | 74.35 | 10.78 |
| No | 2 | 66 | Male | No | 163 | 69 | 26.13 | 90 | 30.7 | 145 | 97 | 4.12 | 1.33 | 1.4 | 1.09 | 5.32 | 5.91 | 7.44 | 1.76 | 17.51 | 125.3 | 13.24 |
| No | 2 | 65 | Male | No | 165 | 62 | 22.77 | 77 | 26.4 | 135 | 78 | 6.46 | 1.23 | 4.37 | 1.53 | 9.97 | 17.12 | 10.84 | 4.8 | 10.92 | 107.31 | 20.3 |
| Yes | 4 | 24 | Male | No | 166 | 75 | 27.22 | 87 | 28.1 | 131 | 78 | 4.28 | 2.36 | 3.34 | 1.32 | 17.52 | 24.06 | 13.52 | 10.53 | 20 | 144.62 | 35.08 |
| Yes | 3 | 29 | Male | No | 159 | 52 | 20.45 | 74 | 24.8 | 150 | 93 | 6.1 | 7.78 | 4.16 | 1.35 | 7.47 | 13.52 | 6.25 | 2.08 | 27.32 | 215.42 | 52.3 |
| Yes | 4 | 30 | Male | No | 180 | 95 | 29.32 | 98 | 28.4 | 140 | 90 | 6.51 | 7.61 | 2.08 | 0.97 | 4.95 | 5.48 | 7.51 | 1.65 | 18.42 | 163.27 | 15.69 |
| Yes | 3 | 31 | Male | Yes | 172 | 72 | 24.34 | 90 | 30.6 | 130 | 80 | 3.94 | 1.55 | 1.87 | 1.37 | 12.7 | 24.62 | 10.86 | 6.13 | 8.4 | 64.15 | 28.3 |
| Yes | 3 | 31 | Female | No | 148 | 44 | 19.95 | 70 | 23.7 | 113 | 63 | 5.02 | 1.63 | 3.2 | 1.08 | 8.37 | 15.04 | 9.12 | 3.39 | 21.44 | 60 | 56.04 |
| Yes | 3 | 32 | Male | No | 167 | 94 | 33.71 | 109 | 34.1 | 145 | 98 | 5.83 | 3.77 | 4.08 | 1.31 | 5.26 | 5.83 | 5.31 | 1.24 | 23.86 | 211.35 | 28.04 |
| Yes | 3 | 33 | Male | No | 161 | 71 | 27.51 | 92 | 33.5 | 140 | 76 | 4.24 | 1.13 | 2.35 | 1.38 | 5.67 | 6.27 | 8.12 | 2.05 | 17.61 | 176.55 | 31.77 |
| Yes | 4 | 34 | Female | No | 153 | 64 | 27.33 | 113 | 37.5 | 149 | 85 | 4.03 | 1.55 | 1.83 | 1.23 | 10.31 | 21.47 | 9.27 | 4.25 | 7.8 | 235.89 | 72.66 |
| Yes | 4 | 36 | Female | No | 149 | 50 | 22.76 | 79 | 28.7 | 138 | 92 | 4.66 | 1.78 | 1.12 | 1.24 | 5.23 | 6.42 | 7.28 | 1.69 | 13.06 | 175.67 | 32.05 |
| Yes | 4 | 36 | Male | No | 170 | 93 | 32.18 | 118 | 35.2 | 153 | 97 | 5.16 | 2.49 | 2.59 | 1.27 | 9.14 | 12.74 | 12.47 | 5.07 | 27.13 | 257.86 | 16.3 |
| Yes | 4 | 37 | Female | Yes | 160 | 65 | 25.39 | 88 | 27.9 | 136 | 81 | 3.94 | 0.84 | 2.42 | 1.14 | 7.88 | 14.25 | 8.04 | 2.82 | 18.62 | 135.98 | 29.04 |
| Yes | 4 | 38 | Male | Yes | 164 | 72 | 26.77 | 88 | 29.3 | 120 | 70 | 5.64 | 4.28 | 1.5 | 0.83 | 8.63 | 17.99 | 17.1 | 6.56 | 24.09 | 146.82 | 16.2 |
| Yes | 4 | 38 | Female | No | 161 | 71 | 27.39 | 91 | 31 | 130 | 84 | 4.02 | 1.16 | 2.3 | 1.19 | 6.66 | 6.66 | 6.16 | 1.82 | 13.53 | 109.42 | 23.4 |
| Yes | 4 | 38 | Female | No | 161 | 71 | 27.39 | 89 | 28.5 | 140 | 90 | 4.02 | 1.16 | 2.3 | 1.19 | 9.09 | 17.06 | 26.41 | 10.67 | 18.32 | 185.32 | 36.41 |
| Yes | 5 | 38 | Male | Yes | 163 | 67 | 25.18 | 88 | 31.5 | 132 | 86 | 3.58 | 4.02 | 0.87 | 0.88 | 5.77 | 6.26 | 8.99 | 2.31 | 25.17 | 253.17 | 37.2 |
| Yes | 3 | 39 | Male | No | 175 | 80 | 26.12 | 88 | 34.1 | 120 | 60 | 4.08 | 1.81 | 2.46 | 0.8 | 4.88 | 5.26 | 8.35 | 1.81 | 24.4 | 149.42 | 18.6 |
| Yes | 4 | 40 | Male | No | 165 | 72 | 26.3 | 86 | 29.2 | 120 | 80 | 5.59 | 1.56 | 4.68 | 0.75 | 5.92 | 7.24 | 11.24 | 2.96 | 28.33 | 157.23 | 16.6 |
| Yes | 3 | 41 | Male | Yes | 164 | 61 | 22.68 | 82 | 30.3 | 132 | 90 | 6.13 | 1.87 | 4.23 | 1.05 | 9.11 | 14.08 | 8.93 | 3.62 | 17.8 | 175.36 | 18.62 |
| Yes | 3 | 41 | Male | No | 169 | 64 | 22.41 | 75 | 24.3 | 115 | 72 | 4.93 | 1.82 | 1.77 | 0.92 | 6.85 | 7.76 | 8.63 | 2.63 | 10.33 | 123.78 | 20.45 |
| Yes | 3 | 41 | Female | No | 162 | 58 | 22.28 | 80 | 26.1 | 117 | 88 | 4.32 | 1.14 | 2.53 | 1.27 | 4.98 | 5.49 | 8.22 | 1.82 | 18.52 | 118.24 | 14.21 |
| Yes | 5 | 42 | Male | No | 165 | 64 | 23.51 | 85 | 29.7 | 120 | 91 | 4.54 | 5.33 | 1.65 | 0.47 | 5.86 | 6.34 | 8.69 | 2.26 | 17.4 | 148.3 | 9.6 |
| Yes | 3 | 42 | Female | No | 160 | 70 | 27.34 | 85 | 25.3 | 132 | 77 | 4.04 | 0.99 | 2.41 | 1.18 | 4.8 | 5.38 | 7.99 | 1.7 | 15.71 | 112.47 | 20.9 |
| Yes | 3 | 43 | Male | No | 172 | 71 | 23.83 | 87 | 27.1 | 118 | 75 | 3.32 | 2.36 | 1.56 | 0.69 | 5.16 | 6.08 | 5.39 | 1.24 | 7.27 | 69.81 | 24.17 |
| Yes | 3 | 43 | Male | Yes | 178 | 95 | 29.98 | 95 | 32.7 | 135 | 70 | 5.46 | 1.97 | 3.42 | 1.1 | 5.06 | 5.69 | 7.7 | 1.73 | 23.86 | 207.34 | 24.5 |
| Yes | 3 | 43 | Female | No | 145 | 46 | 21.93 | 75 | 28.1 | 138 | 89 | 4.69 | 0.69 | 2.5 | 1.88 | 5.78 | 6.38 | 7.78 | 2 | 18.05 | 175.98 | 25.81 |
| Yes | 4 | 43 | Male | Yes | 162 | 70 | 26.67 | 85 | 29.6 | 132 | 88 | 4.48 | 5.55 | 2.64 | 1.2 | 12.14 | 18.13 | 19.54 | 10.54 | 15.82 | 142.83 | 40.05 |
| Yes | 5 | 43 | Female | Yes | 162 | 70 | 26.56 | 93 | 35.2 | 139 | 83 | 4.08 | 1.75 | 2.17 | 1.11 | 5.83 | 6.47 | 8.6 | 2.23 | 23.95 | 196.37 | 55.58 |
| Yes | 3 | 44 | Male | No | 174 | 67 | 22.13 | 83 | 29.2 | 116 | 80 | 2.68 | 1.38 | 1.22 | 0.83 | 9.52 | 16.82 | 8.43 | 3.57 | 15.42 | 118.48 | 39.23 |
| Yes | 3 | 44 | Female | No | 158 | 74 | 29.64 | 113 | 36.5 | 150 | 90 | 5.75 | 2.32 | 3.38 | 1.32 | 5.3 | 5.94 | 8.26 | 1.95 | 20.57 | 253.29 | 8.65 |
| Yes | 3 | 44 | Male | No | 171 | 76 | 25.99 | 85 | 29.3 | 110 | 70 | 3.86 | 1.56 | 0.9 | 0.98 | 5.06 | 5.52 | 9.16 | 2.06 | 21.74 | 174.67 | 20.4 |
| Yes | 5 | 44 | Male | Yes | 173 | 78 | 26.06 | 94 | 35.9 | 168 | 106 | 3.52 | 3.12 | 0.59 | 0.6 | 7.54 | 12.02 | 8.25 | 2.76 | 15.7 | 185.84 | 23.4 |
| Yes | 3 | 44 | Male | No | 171 | 77 | 26.33 | 94 | 30.4 | 150 | 95 | 3.22 | 1.35 | 1.63 | 1.25 | 6.06 | 12.24 | 13.57 | 3.65 | 30.02 | 225.79 | 52.5 |
| Yes | 3 | 44 | Male | No | 174 | 78 | 25.76 | 87 | 28.6 | 116 | 80 | 2.68 | 1.38 | 1.22 | 0.83 | 9.52 | 17.29 | 7.53 | 3.19 | 15.08 | 162 | 82.3 |
| Yes | 3 | 45 | Female | No | 152 | 50 | 21.68 | 73 | 24.7 | 139 | 80 | 3.59 | 2.41 | 1.25 | 1.24 | 5.27 | 5.71 | 10.24 | 2.4 | 12.75 | 103.41 | 12.57 |
| Yes | 5 | 45 | Male | No | 172 | 78 | 26.37 | 89 | 30.7 | 145 | 82 | 8.2 | 7.99 | 1.77 | 0.98 | 6.86 | 7.69 | 7.28 | 2.22 | 18.63 | 133.56 | 23.32 |
| Yes | 4 | 46 | Female | No | 156 | 57 | 23.41 | 79 | 24.7 | 137 | 75 | 4.99 | 2.23 | 3.03 | 0.95 | 5.63 | 6.3 | 6.71 | 1.68 | 6.34 | 81.04 | 19.26 |
| Yes | 5 | 46 | Female | No | 158 | 80 | 31.89 | 108 | 29.4 | 142 | 80 | 4.16 | 2.73 | 1.91 | 1.01 | 5.91 | 6.18 | 4.92 | 1.29 | 19.63 | 237.13 | 23.2 |
| Yes | 4 | 46 | Female | No | 155 | 63 | 26.22 | 83 | 33.6 | 120 | 86 | 4.44 | 1.36 | 2.78 | 1.04 | 5.82 | 6.38 | 8.49 | 2.2 | 33.72 | 175.36 | 23.52 |
| Yes | 3 | 46 | Female | No | 156 | 62 | 25.48 | 84 | 30.4 | 110 | 70 | 4.55 | 1.37 | 2.74 | 1.19 | 7.06 | 10.81 | 8.41 | 2.64 | 19.2 | 132.64 | 25.53 |
| Yes | 4 | 47 | Female | No | 153 | 58 | 24.78 | 78 | 26.5 | 136 | 92 | 5.97 | 6.94 | 0.12 | 0.7 | 7.72 | 16.71 | 10.62 | 3.64 | 25.37 | 144.56 | 13.13 |
| Yes | 3 | 47 | Male | Yes | 164 | 82 | 29.43 | 98 | 35.5 | 140 | 82 | 4.98 | 1.93 | 0.88 | 2.17 | 4.39 | 4.83 | 9.04 | 1.76 | 24.11 | 185.42 | 21.5 |
| Yes | 5 | 47 | Female | Yes | 160 | 62 | 24.22 | 87 | 33.2 | 130 | 84 | 5.2 | 2.52 | 2.84 | 1.21 | 6.26 | 8.62 | 9.29 | 2.58 | 15.1 | 221.08 | 24.6 |
| Yes | 4 | 47 | Male | Yes | 161 | 72 | 27.78 | 89 | 31 | 121 | 77 | 5.03 | 4.66 | 2.71 | 0.91 | 11.68 | 19.92 | 15.27 | 7.93 | 18.4 | 126.74 | 34.76 |
| Yes | 4 | 47 | Female | No | 147 | 57 | 26.52 | 84 | 28.9 | 117 | 73 | 5.09 | 4.13 | 2.97 | 1.14 | 8.21 | 16.91 | 16.47 | 6.01 | 9.45 | 70.23 | 96.6 |
| Yes | 3 | 48 | Female | No | 158 | 69 | 27.64 | 78 | 25.8 | 132 | 85 | 6.16 | 2.12 | 3.73 | 1.27 | 4.89 | 5.35 | 8.57 | 1.86 | 11.13 | 201.67 | 22.64 |
| Yes | 3 | 48 | Male | No | 165 | 54 | 19.88 | 75 | 28.5 | 145 | 91 | 5.47 | 2.55 | 3.07 | 1.24 | 6.86 | 8.33 | 5.29 | 1.61 | 7.92 | 63.33 | 26.44 |
| Yes | 3 | 48 | Male | No | 165 | 70 | 25.71 | 85 | 29.5 | 120 | 79 | 6.19 | 1.74 | 4.45 | 0.95 | 4.95 | 5.41 | 8.67 | 1.91 | 35.75 | 122.83 | 13.5 |
| Yes | 5 | 48 | Female | No | 154 | 62 | 26.14 | 94 | 34.6 | 130 | 80 | 4.18 | 1.86 | 2.41 | 0.92 | 16.45 | 19.54 | 13.69 | 10.01 | 22.78 | 247.48 | 14.4 |
| Yes | 4 | 48 | Male | No | 170 | 77 | 26.64 | 90 | 29.2 | 150 | 80 | 4.52 | 2.5 | 2.43 | 0.95 | 5.2 | 5.83 | 6.74 | 1.56 | 12.06 | 153.78 | 18 |
| Yes | 4 | 48 | Male | No | 163 | 60 | 22.53 | 86 | 27.4 | 136 | 86 | 2.96 | 1.83 | 1.29 | 0.84 | 5.55 | 6.23 | 12.74 | 3.14 | 10.77 | 79.24 | 20.5 |
| Yes | 3 | 48 | Female | No | 152 | 57 | 24.58 | 86 | 31.6 | 117 | 77 | 5.64 | 2.05 | 2.89 | 1.73 | 6.59 | 8.25 | 8.71 | 2.55 | 19.3 | 109.42 | 40.5 |
| Yes | 3 | 49 | Male | Yes | 165 | 60 | 22.04 | 84 | 25.6 | 120 | 80 | 3.74 | 2.32 | 1.24 | 1 | 9.85 | 15.44 | 8.56 | 3.75 | 27.3 | 135.83 | 45.25 |
| Yes | 4 | 49 | Male | No | 159 | 62 | 24.52 | 90 | 32.5 | 123 | 81 | 5.26 | 1.68 | 3.28 | 1.26 | 8.95 | 12.23 | 10.06 | 4 | 18 | 187.33 | 24.3 |
| Yes | 4 | 49 | Male | Yes | 183 | 85 | 25.38 | 93 | 31.8 | 124 | 82 | 3.22 | 2.46 | 4 | 0.78 | 7.35 | 11.53 | 9.65 | 3.15 | 27.48 | 153.57 | 29.06 |
| Yes | 3 | 50 | Male | No | 164 | 61 | 22.68 | 83 | 26.6 | 152 | 90 | 6.13 | 1.87 | 4.23 | 0.95 | 4.19 | 4.95 | 9.24 | 1.72 | 14.7 | 125.54 | 14.91 |
| Yes | 3 | 50 | Male | No | 152 | 52 | 22.46 | 71 | 24.1 | 160 | 102 | 5.58 | 2.14 | 3.12 | 1.21 | 15.3 | 25.44 | 16.55 | 11.25 | 16.05 | 134.42 | 43.95 |
| Yes | 3 | 50 | Female | Yes | 150 | 62 | 27.47 | 86 | 33.6 | 137 | 84 | 5.3 | 1.44 | 3.44 | 1.21 | 5.21 | 5.36 | 7.37 | 1.71 | 13.74 | 154.61 | 14.5 |
| Yes | 3 | 50 | Male | Yes | 168 | 74 | 26.22 | 87 | 36.2 | 130 | 87 | 3.39 | 1.65 | 1.59 | 1.05 | 6.44 | 7.25 | 7.69 | 2.2 | 19.35 | 195.26 | 23.47 |
| Yes | 5 | 50 | Male | Yes | 166 | 71 | 25.77 | 93 | 38.4 | 132 | 74 | 4.35 | 1.83 | 2.61 | 0.91 | 8.16 | 14.42 | 10.16 | 3.68 | 26.74 | 163.32 | 31.83 |
| Yes | 5 | 50 | Male | Yes | 166 | 71 | 25.77 | 95 | 31.2 | 142 | 84 | 4.35 | 1.83 | 2.61 | 0.91 | 6.16 | 11.52 | 6.35 | 1.74 | 13.1 | 134.66 | 34.4 |
| Yes | 4 | 50 | Male | Yes | 165 | 80 | 29.56 | 98 | 26.8 | 127 | 86 | 5.76 | 5.47 | 2.08 | 1.19 | 13.89 | 21.48 | 11.09 | 6.85 | 16.32 | 175.27 | 37.2 |
| Yes | 5 | 50 | Male | Yes | 170 | 94 | 32.53 | 100 | 38.9 | 159 | 105 | 5.68 | 2.06 | 0.86 | 0.84 | 6.72 | 8.62 | 8.67 | 2.59 | 20.63 | 143.39 | 37.45 |
| Yes | 3 | 50 | Female | No | 156 | 64 | 26.3 | 83 | 32 | 145 | 79 | 5.82 | 1.06 | 4.03 | 4.33 | 5.36 | 6.26 | 8.26 | 1.97 | 10.73 | 97.05 | 63.5 |
| Yes | 3 | 51 | Male | No | 163 | 63 | 23.71 | 78 | 28.2 | 110 | 70 | 4.68 | 2.66 | 2.61 | 0.86 | 5.94 | 6.49 | 10.48 | 2.77 | 9.94 | 108.35 | 20.3 |
| Yes | 3 | 51 | Female | No | 158 | 49 | 19.79 | 78 | 23.3 | 131 | 82 | 0.96 | 1.8 | 1.26 | 0.88 | 4.35 | 5.31 | 8.35 | 1.61 | 17.4 | 263.41 | 36.6 |
| Yes | 4 | 51 | Male | No | 162 | 71 | 27.05 | 88 | 32.8 | 160 | 110 | 6.36 | 1.74 | 4.71 | 0.86 | 5.05 | 5.77 | 7.16 | 1.61 | 9.8 | 88.67 | 22.27 |
| Yes | 5 | 51 | Male | No | 165 | 88 | 32.32 | 106 | 35.8 | 143 | 91 | 7.3 | 2.76 | 5.16 | 0.89 | 10 | 16.85 | 9.47 | 4.21 | 22.64 | 205.65 | 23.3 |
| Yes | 3 | 51 | Female | No | 157 | 68 | 27.59 | 91 | 31.7 | 125 | 81 | 5.88 | 1.55 | 4.11 | 1.07 | 5.78 | 6.49 | 6.54 | 1.68 | 10.88 | 103.55 | 23.43 |
| Yes | 4 | 51 | Female | No | 160 | 75 | 29.3 | 95 | 30.5 | 138 | 82 | 5.74 | 1.81 | 3.61 | 1.3 | 5.33 | 6.17 | 9.35 | 2.21 | 19.42 | 201.45 | 23.57 |
| Yes | 3 | 52 | Male | Yes | 172 | 63 | 21.3 | 76 | 26.3 | 132 | 93 | 5.21 | 2.12 | 2.89 | 1.31 | 11.95 | 21.89 | 12.98 | 6.89 | 13.42 | 152 | 18.23 |
| Yes | 3 | 52 | Female | No | 155 | 50 | 20.73 | 89 | 35.4 | 136 | 86 | 5.3 | 2.02 | 3.17 | 1.76 | 5.07 | 5.87 | 6.91 | 1.56 | 16.6 | 67.6 | 8.42 |
| Yes | 4 | 52 | Male | Yes | 164 | 81 | 30.11 | 100 | 30 | 131 | 92 | 5.26 | 3.43 | 2.12 | 1.02 | 14.28 | 21.5 | 9.45 | 6 | 21.53 | 206.55 | 18.98 |
| Yes | 4 | 52 | Female | No | 155 | 63 | 26.26 | 81 | 29.2 | 142 | 73 | 5.8 | 1.46 | 4.6 | 0.99 | 6.71 | 7.29 | 9.32 | 2.78 | 14.85 | 116.12 | 24.1 |
| Yes | 5 | 52 | Female | No | 149 | 57 | 25.45 | 79 | 26.3 | 128 | 85 | 3.67 | 2.08 | 1.56 | 1.16 | 8.89 | 12.56 | 12.86 | 5.08 | 19.94 | 145.03 | 27.4 |
| Yes | 4 | 52 | Female | No | 159 | 78 | 30.85 | 103 | 34 | 147 | 98 | 5.04 | 2.47 | 1.79 | 0.79 | 12.04 | 19.32 | 12.7 | 6.8 | 20.6 | 206.37 | 31.23 |
| Yes | 5 | 52 | Male | No | 158 | 65 | 26.04 | 95 | 33.6 | 126 | 86 | 4.95 | 1.73 | 1.24 | 2.33 | 10.77 | 19.36 | 13.1 | 6.27 | 20.55 | 77.68 | 35.29 |
| Yes | 3 | 53 | Female | No | 156 | 51 | 21.13 | 76 | 25.1 | 131 | 81 | 6.09 | 1.77 | 3.87 | 1.12 | 5.18 | 5.71 | 10.82 | 2.49 | 7.92 | 94.67 | 14.21 |
| Yes | 3 | 53 | Female | Yes | 156 | 51 | 21.13 | 77 | 23 | 141 | 81 | 6.09 | 1.77 | 3.87 | 1.42 | 10.6 | 15.75 | 10.19 | 4.8 | 8.7 | 76.21 | 17.81 |
| Yes | 3 | 53 | Male | No | 168 | 65 | 23.03 | 81 | 28.1 | 95 | 60 | 4.67 | 3.38 | 1.89 | 0.94 | 7.72 | 10.58 | 9.61 | 3.3 | 8.31 | 84.5 | 26.4 |
| Yes | 3 | 53 | Male | No | 162 | 63 | 23.93 | 82 | 25.8 | 97 | 55 | 4.66 | 2.56 | 2.35 | 1.15 | 9.46 | 20.4 | 18.69 | 7.86 | 14.07 | 88.3 | 29.52 |
| Yes | 5 | 53 | Female | No | 158 | 69 | 27.64 | 95 | 32.6 | 134 | 87 | 4.71 | 2.53 | 1.81 | 0.75 | 11.2 | 17.27 | 12.74 | 6.34 | 18.98 | 237.47 | 28.5 |
| Yes | 3 | 55 | Female | No | 159 | 63 | 24.92 | 82 | 27.5 | 138 | 78 | 5.16 | 1.91 | 2.63 | 1.66 | 4.5 | 5.38 | 4.9 | 0.98 | 34 | 114.93 | 13.54 |
| Yes | 3 | 55 | Female | Yes | 153 | 68 | 29.05 | 87 | 30.6 | 119 | 63 | 5.29 | 1.58 | 2.83 | 1.42 | 6.63 | 8.93 | 10.2 | 3.01 | 10.73 | 174.47 | 13.7 |
| Yes | 3 | 55 | Female | No | 155 | 64 | 26.6 | 81 | 30.6 | 128 | 66 | 3.28 | 3.08 | 0.97 | 0.91 | 5.13 | 6.07 | 8.48 | 1.93 | 10.9 | 115.3 | 14.5 |
| Yes | 3 | 55 | Male | No | 165 | 76 | 27.92 | 93 | 36.4 | 124 | 78 | 5.12 | 2.03 | 2.9 | 1.3 | 6.04 | 7.89 | 10.34 | 2.78 | 24.06 | 174.74 | 25.73 |
| Yes | 5 | 55 | Female | No | 153 | 58 | 24.78 | 97 | 33.2 | 157 | 102 | 6 | 2.14 | 4.03 | 1 | 7.88 | 14.82 | 9.24 | 3.24 | 21.85 | 205.11 | 26.3 |
| Yes | 3 | 55 | Female | No | 151 | 47 | 20.75 | 81 | 27.5 | 133 | 82 | 4.8 | 1.79 | 2.38 | 1.41 | 4.53 | 6.81 | 4.69 | 0.94 | 10 | 44 | 28.48 |
| Yes | 3 | 56 | Female | Yes | 155 | 61 | 25.31 | 86 | 29.1 | 143 | 76 | 4.12 | 0.8 | 2.34 | 1.42 | 6.37 | 6.92 | 7.25 | 2.05 | 28.83 | 252.55 | 9.4 |
| Yes | 3 | 56 | Male | No | 165 | 83 | 30.49 | 93 | 35.6 | 136 | 83 | 4.64 | 1.27 | 0.73 | 1.35 | 4.74 | 5.44 | 8.06 | 1.7 | 15.01 | 190.53 | 9.78 |
| Yes | 3 | 56 | Female | No | 155 | 61 | 25.31 | 90 | 28.5 | 133 | 76 | 4.12 | 0.8 | 2.34 | 1.42 | 5.71 | 6.42 | 7.51 | 1.91 | 19.74 | 144.37 | 13.54 |
| Yes | 4 | 56 | Female | Yes | 162 | 78 | 30.02 | 105 | 34.7 | 119 | 73 | 5.11 | 4.22 | 2.25 | 0.85 | 8.87 | 14.24 | 11.82 | 4.66 | 24.33 | 235.6 | 22.65 |
| Yes | 3 | 56 | Female | No | 161 | 68 | 26.23 | 88 | 32.4 | 113 | 69 | 5.26 | 4.41 | 2.06 | 2.26 | 6.34 | 8.24 | 9.45 | 2.66 | 18.8 | 213.6 | 91.96 |
| Yes | 3 | 57 | Female | No | 160 | 56 | 21.88 | 75 | 25.4 | 126 | 87 | 4.16 | 1.8 | 2.43 | 0.91 | 5.22 | 6.28 | 8.37 | 1.94 | 17.62 | 147.21 | 14.21 |
| Yes | 3 | 57 | Female | Yes | 159 | 58 | 23.02 | 77 | 28.4 | 140 | 93 | 5.38 | 1.78 | 3.16 | 1.21 | 5.31 | 6.14 | 8.05 | 1.9 | 12.3 | 88.49 | 19.3 |
| Yes | 4 | 57 | Female | No | 150 | 57 | 25.33 | 83 | 27.3 | 133 | 80 | 5.2 | 1.23 | 2.44 | 1.16 | 5.35 | 6.25 | 7.34 | 1.75 | 6.89 | 66.31 | 9.28 |
| Yes | 3 | 57 | Female | No | 155 | 69 | 28.72 | 92 | 32.6 | 126 | 81 | 5.39 | 2.26 | 4.02 | 1.6 | 14.72 | 24.93 | 14.88 | 9.73 | 14.84 | 84.31 | 13.5 |
| Yes | 4 | 57 | Female | No | 160 | 56 | 21.88 | 76 | 26.8 | 150 | 90 | 4.16 | 1.8 | 2.43 | 0.91 | 9.45 | 14.75 | 12.76 | 5.36 | 19.6 | 195.43 | 19.4 |
| Yes | 3 | 58 | Male | Yes | 170 | 70 | 24.15 | 82 | 27.7 | 150 | 86 | 3.28 | 2.16 | 1.07 | 0.93 | 4.96 | 5.63 | 10.9 | 2.4 | 17.32 | 92.31 | 18.9 |
| Yes | 4 | 58 | Female | No | 152 | 52 | 22.68 | 82 | 26.8 | 124 | 81 | 4.93 | 2.48 | 2.56 | 1.24 | 5.79 | 6.48 | 10.79 | 2.78 | 14.96 | 66.6 | 22.4 |
| Yes | 3 | 59 | Female | No | 152 | 59 | 25.71 | 87 | 28.6 | 124 | 79 | 5.26 | 2.24 | 3.07 | 1.17 | 5.2 | 5.68 | 6.24 | 1.44 | 10.85 | 126.69 | 8.56 |
| Yes | 3 | 59 | Male | Yes | 165 | 79 | 29.02 | 96 | 27.5 | 150 | 100 | 4.58 | 3.01 | 2.06 | 1.12 | 4.72 | 5.49 | 8.36 | 1.75 | 19.52 | 158.53 | 17.78 |
| Yes | 5 | 60 | Male | No | 174 | 81 | 26.75 | 86 | 31 | 130 | 70 | 3.39 | 1.7 | 1.8 | 0.82 | 7.29 | 12.82 | 7.29 | 2.36 | 20.61 | 148.64 | 15.4 |
| Yes | 3 | 60 | Female | No | 149 | 55 | 24.59 | 82 | 28.1 | 136 | 80 | 5.23 | 1.4 | 3.03 | 1.56 | 8.1 | 14.56 | 8.5 | 3.06 | 18.05 | 175.98 | 16.7 |
| Yes | 3 | 60 | Male | No | 165 | 62 | 22.77 | 85 | 27.7 | 130 | 90 | 6.45 | 4.06 | 3.3 | 1.3 | 5.12 | 6.34 | 8.69 | 1.98 | 23.63 | 123.72 | 19.26 |
| Yes | 3 | 60 | Male | No | 178 | 92 | 29.04 | 97 | 30.4 | 128 | 86 | 5.76 | 2.13 | 3.6 | 1.19 | 5.11 | 5.84 | 11.14 | 2.53 | 19.64 | 179.51 | 24 |
| Yes | 4 | 60 | Male | Yes | 168 | 77 | 27.28 | 86 | 28.8 | 132 | 82 | 6.34 | 2.44 | 3.6 | 1.63 | 8.26 | 16.24 | 21.11 | 7.75 | 7.14 | 79.33 | 33.67 |
| Yes | 3 | 61 | Male | Yes | 167 | 60 | 21.51 | 72 | 23.2 | 120 | 80 | 3.34 | 2.29 | 1.6 | 0.7 | 5.79 | 6.19 | 9.24 | 2.38 | 14.2 | 117.73 | 14.6 |
| Yes | 3 | 61 | Male | No | 170 | 71 | 24.57 | 81 | 24.6 | 132 | 73 | 4.02 | 1.99 | 2.11 | 1.01 | 5.73 | 6.5 | 6.81 | 1.73 | 18.24 | 124.08 | 30.95 |
| Yes | 3 | 61 | Female | Yes | 155 | 66 | 27.78 | 94 | 32.9 | 134 | 77 | 4.88 | 0.99 | 2.89 | 1.54 | 6.28 | 14.25 | 10.26 | 2.86 | 28.5 | 236.64 | 29.4 |
| Yes | 4 | 62 | Female | No | 159 | 58 | 21.3 | 74 | 26.1 | 143 | 84 | 5.72 | 2.91 | 2.66 | 1.28 | 5.88 | 7.71 | 7.26 | 1.9 | 8.2 | 158.27 | 13.5 |
| Yes | 3 | 62 | Male | No | 164 | 61 | 22.68 | 83 | 27.1 | 131 | 91 | 6.04 | 4.56 | 3.27 | 0.97 | 5.06 | 5.44 | 8.72 | 1.96 | 10.52 | 120.83 | 18.65 |
| Yes | 5 | 62 | Female | No | 147 | 53 | 24.53 | 90 | 34.7 | 140 | 82 | 4.85 | 3.63 | 2.25 | 0.95 | 6.34 | 7.32 | 6.37 | 1.79 | 15.9 | 184.2 | 13.7 |
| Yes | 3 | 62 | Female | No | 150 | 51 | 22.62 | 83 | 24.7 | 133 | 70 | 6.28 | 1.56 | 3.4 | 1.77 | 5.61 | 6.47 | 7.39 | 1.84 | 5.08 | 55.08 | 15.25 |
| Yes | 3 | 62 | Male | No | 170 | 83 | 28.72 | 87 | 26.4 | 146 | 87 | 5.61 | 0.9 | 3.8 | 1.4 | 13.31 | 21.64 | 15.12 | 8.94 | 10.6 | 98.67 | 15.69 |
| Yes | 3 | 62 | Male | No | 170 | 63 | 21.87 | 87 | 30.2 | 126 | 87 | 5.61 | 0.9 | 3.8 | 1.4 | 5.82 | 5.81 | 9.14 | 2.36 | 10.73 | 163.29 | 24.32 |
| Yes | 5 | 62 | Female | No | 155 | 61 | 25.18 | 84 | 27.9 | 138 | 72 | 3.16 | 1.71 | 1.08 | 1.3 | 5.82 | 6.48 | 9.33 | 2.41 | 16.32 | 120.4 | 24.75 |
| Yes | 4 | 62 | Female | No | 150 | 70 | 31.11 | 93 | 30.4 | 135 | 83 | 5.41 | 2.16 | 2.68 | 1.31 | 17.48 | 23.09 | 12.04 | 9.35 | 14.7 | 153.65 | 28 |
| Yes | 5 | 62 | Female | No | 154 | 57 | 24.03 | 86 | 25.2 | 165 | 90 | 5.27 | 2.56 | 2.87 | 1.24 | 8.02 | 12.82 | 10.2 | 3.64 | 12.83 | 178.41 | 34.2 |
| Yes | 4 | 63 | Female | No | 151 | 60 | 26.45 | 90 | 35.9 | 137 | 75 | 5.99 | 1.14 | 3.58 | 1.19 | 6.03 | 8.34 | 9.44 | 2.53 | 10.2 | 173.22 | 24.23 |
| Yes | 4 | 64 | Female | No | 147 | 43 | 20.13 | 78 | 25.6 | 131 | 72 | 5.73 | 1.94 | 3.59 | 1.26 | 10.66 | 18.07 | 8.3 | 3.93 | 11 | 57.6 | 20.79 |
| Yes | 4 | 64 | Female | No | 157 | 63 | 25.64 | 94 | 26.8 | 178 | 97 | 4.21 | 1.2 | 2.53 | 1.13 | 16.24 | 22.3 | 12.37 | 8.93 | 13.42 | 115.62 | 38.49 |
| Yes | 3 | 64 | Male | Yes | 151 | 47 | 20.75 | 81 | 27.5 | 120 | 70 | 4 | 1.7 | 1.71 | 1.06 | 7.15 | 9.34 | 9.33 | 2.96 | 14.24 | 155.66 | 44.1 |
| Yes | 3 | 64 | Female | Yes | 141 | 53 | 26.8 | 84 | 28.4 | 135 | 68 | 4.9 | 1.84 | 2.69 | 1.56 | 15.13 | 21.7 | 10.57 | 7.11 | 9.17 | 64 | 58.31 |
| Yes | 3 | 65 | Male | No | 165 | 76 | 27.92 | 88 | 30.5 | 124 | 78 | 5.12 | 2.03 | 2.9 | 1.3 | 14.18 | 18.61 | 10.22 | 6.44 | 14.3 | 82.95 | 40.5 |
| Yes | 3 | 66 | Male | No | 157 | 50 | 20.41 | 67 | 24.6 | 128 | 88 | 5.68 | 1.72 | 3.57 | 1.16 | 12.18 | 16.49 | 11.93 | 6.46 | 7.92 | 85.33 | 19.86 |
| Yes | 3 | 66 | Female | No | 149 | 50 | 22.76 | 82 | 26.5 | 148 | 82 | 3.67 | 1.92 | 1.12 | 1.68 | 4.75 | 5.71 | 5.84 | 1.23 | 18.32 | 132.76 | 14.32 |
| Yes | 4 | 67 | Male | No | 168 | 75 | 26.57 | 94 | 25.9 | 118 | 73 | 6.64 | 1.81 | 4.8 | 0.98 | 5.81 | 6.38 | 10.18 | 2.63 | 9.37 | 165.1 | 23.53 |
| Yes | 4 | 67 | Female | No | 149 | 58 | 26.12 | 94 | 28.5 | 138 | 84 | 4.2 | 2.17 | 2.31 | 0.95 | 4.77 | 5.49 | 8.28 | 1.76 | 20.42 | 165.77 | 23.86 |
| Yes | 5 | 67 | Female | No | 154 | 63 | 26.91 | 91 | 34.2 | 142 | 82 | 5.22 | 2.16 | 3.02 | 1.07 | 7.66 | 13.62 | 8.76 | 2.98 | 17.34 | 116.85 | 34.44 |
| Yes | 4 | 67 | Male | Yes | 168 | 75 | 26.57 | 90 | 32.1 | 140 | 85 | 6.64 | 1.81 | 4.8 | 1.02 | 6.07 | 10.52 | 8.32 | 2.24 | 16.23 | 135.68 | 38.5 |
| Yes | 3 | 67 | Female | No | 153 | 58 | 24.94 | 79 | 22.6 | 132 | 76 | 5.42 | 1.51 | 2.18 | 1.29 | 4.25 | 5.47 | 5.16 | 0.97 | 13.84 | 143.09 | 51.8 |

MetS: Metabolic Syndrome; BMI: Body mass index; Fat%: Percentage of body fat; SBP: Systolic blood pressure; DBP: Diastolic blood pressure; TC: Total cholesterol; TG: Triglycerides; LDLC: Low-density lipoprotein cholesterol; HDLC: High-density lipoprotein cholesterol; FPG: Fasting plasma glucose; 2h PG: 2h plasma glucose; FINS: Fasting insulin; HOMA-IR: Homeostasis model assessment of insulin resistance; IL-6: Interleukin-6; MCP-1; Monocyte chemotactic protein-1.
